# A translational physiologically-based pharmacokinetic model for MMAE-based antibody-drug conjugates

**DOI:** 10.1007/s10928-025-09978-3

**Published:** 2025-05-05

**Authors:** Hsuan-Ping Chang, Dhaval K. Shah

**Affiliations:** Department of Pharmaceutical Sciences, School of Pharmacy and Pharmaceutical Sciences, The State University of New York at Buffalo, 455 Pharmacy Building, Buffalo, NY 14214-8033 USA

**Keywords:** Antibody-drug conjugate (ADC), Monomethyl auristatin E (MMAE), Tissue distribution, Pharmacokinetics, Physiologically-based pharmacokinetic (PBPK) model, Interspecies scaling, Clinical pharmacokinetics

## Abstract

The objective of this work was to develop a translational physiologically-based pharmacokinetic (PBPK) model for antibody-drug conjugates (ADCs), using monomethyl auristatin E (MMAE)-based ADCs. A previously established dual-structured whole-body PBPK model for MMAE-based ADCs in mice was scaled to higher species (i.e., rats and monkeys) and humans. Species-specific physiological and drug-related parameters for the payload and antibody backbone of ADCs were obtained from literature. Parameters associated with payload release, including the deconjugation rate, were optimized using an allometric scaling approach, and antibody degradation rate was adjusted to account for the enhanced clearance of ADCs due to conjugation across different species. The translational PBPK model predicted the PK profiles for various ADC analytes in rats, monkeys, and humans reasonably well. The optimized PBPK model suggested decreased rate of deconjugation for ADCs in higher species, whereas the effects of payload conjugation on ADC clearance were more pronounced in higher species and humans. The translational PBPK model presented here may enable prediction of different ADC analyte PK at the site-of-action, offering valuable insights for the development of exposure-response relationships for ADCs. The modeling framework presented here can also serve as a platform for the development of PBPK model for other ADCs.

**Supplementary Information:**

The online version contains supplementary material available at 10.1007/s10928-025-09978-3.

## Introduction

Antibody-drug conjugates (ADCs) are a promising anticancer drug molecules that can significantly improve the outcome of cancer treatment. However, their clinical success is limited by the narrow therapeutic window. In order to improve the therapeutic window of ADCs it is important to establish reliable relationships between the exposure and pharmacology (i.e., toxicities and efficacy) of ADCs. Previous exposure-toxicity analyses for brentuximab vedotin, which relied on plasma pharmacokinetics (PK) of ADCs, failed to reach a consensus on which ADC analytes and exposure matrices best drive toxicities [1, 2]. This suggests that plasma PK alone may not be sufficient for establishing exposure-toxicity relationships for ADCs, and comprehensive understanding of tissue PK may be needed [3]. However, the complex molecular nature of ADCs that consist of both large and small molecules complicates their PK analysis, which necessitates multiple assay formats to measure various analytes. Quantification of ADCs in different tissue matrices poses additional challenge [4], and current methodologies are inadequate for assessing whole-body distribution of ADC analytes in humans. As such, there is a need to develop tools that can predict whole-body PK of ADC analytes in the clinic, and can be used to establishment reliable exposure-response relationships. Here we have proposed a translational physiologically-based PK (PBPK) model developed using monomethyl Auristatin E (MMAE)-based ADCs as that tool that can be used to characterize and predict whole-body PK of ADCs.

In the past, Chen et al. [5] and Semineni et al. [6] have developed a minimal PBPK model for MMAE-based ADCs using the Simcyp simulator [7] to assess drug-drug interaction (DDI) risks in humans. Although the model accurately characterized the plasma PK of conjugated and unconjugated payloads observed in clinical studies, its minimal PBPK structure—limited to plasma without tissue compartments and relying on an empirical adjustment—prevented it from predicting tissue PK and assessing the potential risks of free payload exposure in healthy tissues. More recently, another PBPK model for MMAE-based ADCs, also developed using the Simcyp simulator, aimed to assess the DDI potential of enfortumab vedotin [8]. While this updated model incorporated a full PBPK model structure for unconjugated MMAE, it remained confined to a minimal PBPK model structure for the conjugated antibody (mAb) component. As a result, the model fell short in describing tissue PK for the conjugates, thereby limiting its capacity to study toxicities potentially resulting from healthy tissues expressing the target (i.e., on-target off-tumor toxicities). Thus, the inability of these models to predict tissue PK across different ADC analytes limit their utility in establishing reliable exposure-toxicity relationships for ADCs. Cilliers et al. developed a multi-scale PBPK model capable of monitoring systemic and tissue-level ADC distribution [9]. The model integrated a whole-body PBPK framework with a tissue-level distribution model based on Krogh cylinder geometry for tumors [10]. The model was able to characterize the biodistribution of fluorescently labeled Trastuzumab emtansine (T-DM1). Although the model could assess multiple factors—such as target antigen, drug-antibody ratio (DAR), linker stability, mAb, and payload—and evaluate their impact on ADC efficacy, it could only predict the biodistribution of mAb component of an ADC. This limitation hampers the model’s ability to accurately investigate the toxicity aspects of ADCs.

We have previously developed a whole-body PBPK model for T-DM1, integrating a small molecule PBPK model and a mAb PBPK model. Whole-body disposition data of [^3^H]DM1 in rats informed the development of the small molecule model, and the platform PBPK model for mAbs characterized the PK of the conjugated mAb of T-DM1 in rats [11]. These models were linked through deconjugation and degradation processes. The model was able to predict the biodistribution of radiolabeled T-[^3^H]DM1 in rats reasonably well and was further translated to humans for predicting the clinical PK of T-DM1. This translation involved using the human mAb PBPK model to characterize PK of the conjugated mAb of T-DM1, employing allometric scaling to estimate human clearance of DM1 catabolites, and utilizing monkey PK data to predict DM1 deconjugation in humans. While the model enabled predicting the plasma PK of conjugated and total mAbs of T-DM1, it showed a slight prediction bias for the plasma PK of DM1 in humans. This bias might stem from the simplifying assumption that all released payloads exhibit similar PK properties, while the major catabolites of T-DM1, including DM1, lysine-MCC-DM1, and MCC-DM1, may follow distinct PK behaviors [12]. Li et al. presented a poster outlining the development of a whole-body PBPK model for MMAE-conjugated ADCs, based on radiolabeled data in rats [13]. This model incorporated DAR-dependent deconjugation and an additional empirical DAR-dependent plasma clearance mechanism. The model was designed to describe individual DAR species; however, it lacked validation data to differentiate deconjugation rates or plasma clearance among different DAR species. Furthermore, the poster provided insufficient details regarding the development of the small molecule PBPK model, which could compromise its utility in establishing robust exposure-toxicity relationships for ADCs.

We have also developed a whole-body PBPK model for MMAE-based ADCs, utilizing whole-body PK data in mice [14]. This model integrated a platform PBPK model for mAbs [15] with a published PBPK model for MMAE [16], linked through non-specific deconjugation and proteolytic degradation processes at the tissue level. The model was developed based on several assumptions: the total mAb of ADC and the naked mAb backbone share similar intracellular processes; unconjugated MMAE exhibits PK properties similar to those of naked MMAE; ADCs degraded in endosomal compartments release payloads equivalent to the average DAR value at a given time; and a first-order DAR-dependent deconjugation process occurs uniformly across all tissues. This model was capable of predicting the biodistribution of MMAE-based ADCs in mice with reasonable accuracy. To extend the applicability of this model to other preclinical species and for the clinical translation of ADCs, here we have scaled up the model to higher species and humans using published datasets. This translated human PBPK model offers an opportunity for characterizing and predicting the tissue exposure of different ADC analytes in the clinic, which may facilitate the establishment of more reliable exposure-response relationships for ADCs going forward.

## Methods

### Data collection

To facilitate the development of a translational PBPK model, literature-reported datasets describing the PK of MMAE-based ADCs in rats, monkeys, and humans were collected. A comprehensive literature search was conducted using databases such as PubMed and Google Scholar, conference proceedings, abstracts, posters, regulatory submissions from the FDA and EMA, and other web-based resources. Search keywords included MMAE, vc-MMAE, vendotin, MMAE-based ADC, and other related terms. Previously, we developed an ADC PBPK model in mice using whole-body biodistribution data for vc-MMAE ADCs [16]. Therefore, the data collected for the development of the translational PBPK model across species exclusively included ADCs with vc-MMAE as the linker-payload and a stochastic conjugation method. Studies and PK data were selected based on predetermined criteria: the inclusion of ADCs utilizing vc-MMAE as a linker payload and stochastic conjugation method, the availability of PK profiles for at least one of the four ADC analytes (total mAb, conjugated mAb, unconjugated MMAE, conjugated MMAE), and linear PK at the dosages tested. Studies involving ADCs that employed different conjugation methods or linker chemistries were excluded. Upon identification of eligible studies, plasma and, if available, tissue PK data for all reported ADC analytes were collected. These data were digitized using WebPlotDigitizer [17], and the respective doses and units of concentrations for each PK profile were recorded to facilitate subsequent data analysis.

### Data analysis

The collected PK profiles were dose-normalized to 10 mg/kg for rats, 5 mg/kg for monkeys, and 2.4 mg/kg for humans. The doses selected for normalization are based on the most frequently tested doses in the published studies for rats and monkeys, and most commonly used dosing regimen in the expansion cohorts of Phase I trials for vc-MMAE ADCs (2.4 mg/kg given every three weeks, Q3W) [18]. Data from individual ADCs after dose-normalization were pooled for subsequent analysis. This approach is supported by our previous study, which showed that dose-normalized PK profiles across distinct vc-MMAE ADCs were generalizable and could be described using a unified PK model [19]. The average DAR vs. time profile for MMAE-based ADCs in plasma was calculated using Eq. 1.

1 $$\:{\text{D}\text{A}\text{R}}_{\text{t}}=\frac{{\text{C}}_{\text{p}}^{\text{a}\text{c}\text{M}\text{M}\text{A}\text{E}}}{{\text{C}}_{\text{p}}^{\text{m}\text{A}\text{b}}}$$

Above, $\:{\text{D}\text{A}\text{R}}_{\text{t}}$ represents the average DAR at time t and $\:{\text{C}}_{\text{p}}^{\text{a}\text{c}\text{M}\text{M}\text{A}\text{E}}$ and $\:{\text{C}}_{\text{p}}^{\text{m}\text{A}\text{b}}$ denote the mean plasma concentrations of conjugated MMAE and total mAb, respectively, calculated using the pooled dose-normalized data at each time point.

### PBPK model structure

**Fig. 1:**
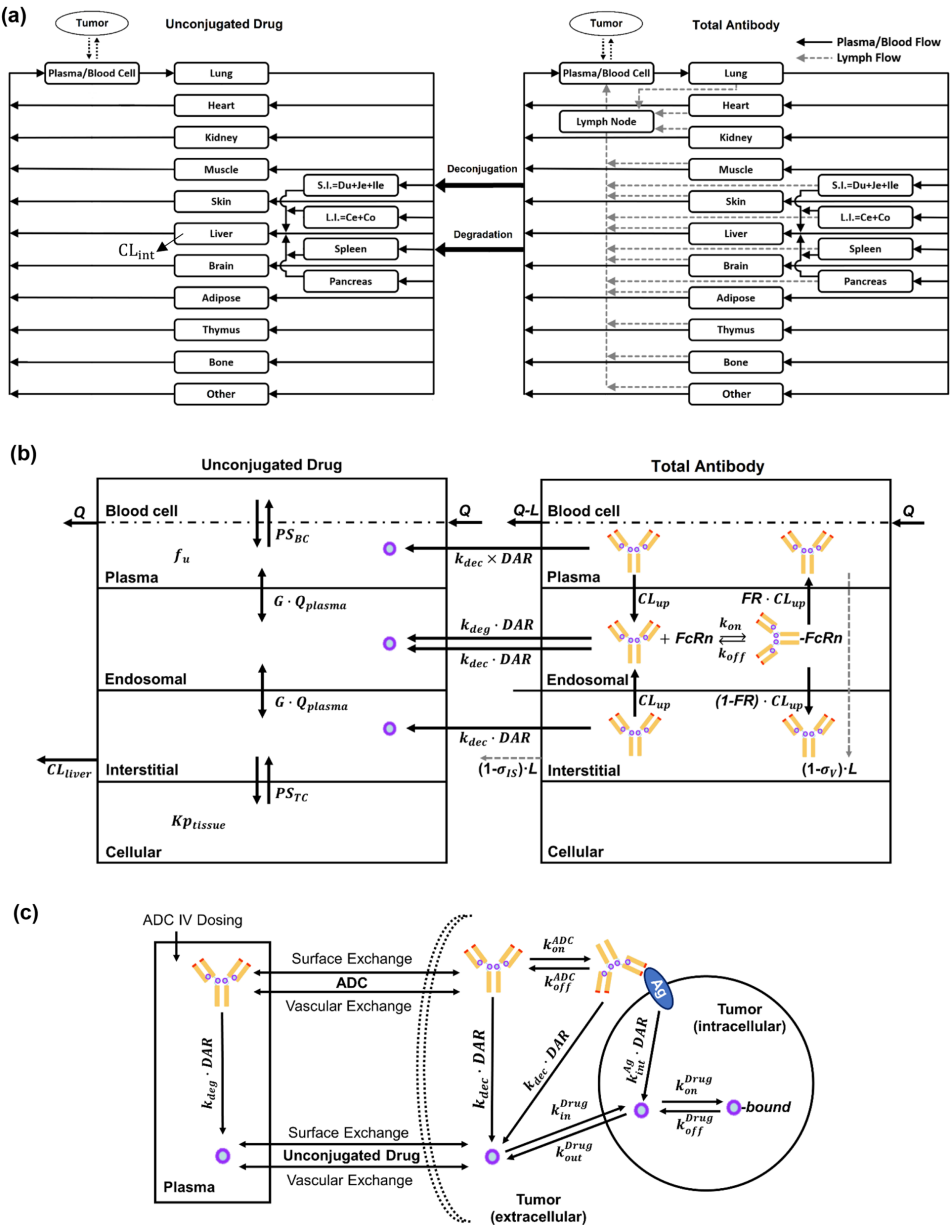
PBPK model structure for (**a**) whole-body and (**b**) tissue-level disposition of MMAE-based ADCs. The model consists of an antibody and a small molecular PBPK model connected via deconjugation and degradation processes. All organs are represented by a rectangular compartment and connected in an anatomical manner with blood flow (solid arrows) and lymphatic flow (dashed arrows). Each tissue within the model, except the blood and lymph node compartment, is further divided into plasma, blood cell, endosomal, interstitial, and cellular sub-compartments. For a detailed description of the symbols and drug disposition processes, please refer to the Model Structure section in the Methods; (**c**) Schematics of cell-level tumor disposition model for ADC

Figure 1 illustrates the structure of the whole-body PBPK model for vc-MMAE-based ADCs, developed by mechanistically integrating previously established PBPK models for MMAE [16] and mAbs [15] in a mechanistic manner.

The left panel of Fig. 1a and b displays the PBPK model used to describe the whole-body and tissue-level disposition of unconjugated MMAE generated from the ADC. The structure and assumptions of this model have been previously reported [16]. Briefly, the model included 16 tissue compartments such as blood, lung, heart, kidney, muscle, skin, liver, brain, fat, thymus, bone, small intestine, large intestine, spleen, pancreas, and an “other” compartment, as well as a tumor compartment. These tissue compartments were anatomically arranged and interconnected through blood flow. Each tissue compartment was divided into sub-compartments: plasma, blood cells, endothelial cells, interstitial space, and cellular space. Hepatic clearance within the interstitial space was assumed to be the primary elimination pathway for MMAE [1, 5, 16, 20]. Rapid distribution of MMAE among plasma, endothelial cells, and interstitial space was also assumed. Permeability-limited distributions between plasma and blood cells, as well as between interstitial and cellular spaces were modeled using permeability coefficients (PS) for each tissue. Partitioning of MMAE into blood cells or cellular spaces was characterized using partition coefficient ($\:{\text{K}}_{\text{p}}$) values, derived from biodistribution data for free MMAE in mice.

The right panel of Fig. 1a and b outlines the PBPK model that describes the whole-body and tissue-level disposition of the mAb component of an ADC. The model structure and features have been detailed in previous work [15]. Briefly, the model incorporated 17 tissues and a tumor compartment, and each tissue was divided into the same five sub-compartments as the PBPK model for MMAE. Once mAbs entered the tissue vasculature, they were transported into the endosomal space of endothelial cells via pinocytosis. There, the mAb component can bind to FcRn, which can facilitate either transcytosis or recycling back into the systemic circulation. FcRn-unbound mAbs in the endosomal space would go to late lysosome for degradation.

The large and small molecule PBPK models were connected through degradation and deconjugation processes. Each ADC molecule degraded in the endosomal compartment was assumed to release a certain number of payloads equivalent to the ADC DAR value at the given time. Furthermore, the conjugated drug was presumed to undergo non-specific, DAR-dependent deconjugation in plasma, interstitial, and endosomal sub-compartments in all tissues. The average DAR of ADCs (Eq. 1) was used to represent the overall DAR of the ADC in the entire body. The deconjugation rate constant ($\:{\text{k}}_{\text{d}\text{e}\text{c}}$) was optimized using an empirical equation (Eq. 2) developed in mice [14], which included a scaling factor (SF) to capture the observed biphasic DAR decline profile across species, as characterized by Eq. 3. In Eq. 2, the parameter values of α, β, F, and τ were 0.0806, 0.0102, 0.282, and 5.68, respectively. For interspecies scaling of the SF, a simple allometric scaling with an exponent of -0.25 was applied, where SF = (body weight ratio)^−0.25^. The released MMAE was assumed to have the same PK properties as MMAE dosed in the free form. The equations of translational PBPK model for MMAE-based ADC are provided in Supplementary Appendix 1.

2 $$\:{\text{k}}_{\text{d}\text{e}\text{c}}=({\upalpha\:}-\frac{\left({\upalpha\:}-{\upbeta\:}\right)\times\:{\text{D}\text{A}\text{R}}^{-{\uptau\:}}}{\text{F}+{\text{D}\text{A}\text{R}}^{-{\uptau\:}}})\times\:\text{S}\text{F}$$

3 $$\:\frac{\text{d}\text{D}\text{A}\text{R}}{\text{d}\text{t}}=-{\text{k}}_{\text{d}\text{e}\text{c}}\times\:\text{D}\text{A}\text{R}$$

### Model parameters

PK parameters related to the mAb or MMAE components of ADCs were sourced from the literature for rats, monkeys, and humans [15, 16]. The physiological parameters used in the translational PBPK model for MMAE-based ADC in rats, monkeys, and humans are provided in Tables S1, S2, and S3, respectively [15]. A glossary of literature-derived parameters used in the ADC PBPK model is provided in Table S4 [14, 21]. For the MMAE component, parameters such as plasma protein binding ($\:{\text{f}}_{\text{u}\text{p}}$), hepatic intrinsic clearance ($\:{\text{C}\text{L}}_{\text{i}\text{n}\text{t}}$), and red blood cell partitioning varied by species [20, 22, 23]. The reported MMAE $\:{\text{f}}_{\text{u}\text{p}}$ was 81.4% (range 72.0–90.8%) in rats and 75.1% (67.9–82.2%) in humans [22, 23]. In monkeys, reported values diverged significantly between two sources, either 18% (17.1–18.9%) [22] or 65.3% (61.4–69.2%) [23], and thus both values for monkeys were assessed in model simulations to determine the best fit with observed data. Red blood cell partitioning for MMAE was approximately 4 in rats, 3 in monkeys, and ranged from 1.34 to 1.65 in humans [20, 22, 23]. The $\:{\text{K}}_{\text{p}}$ value for MMAE in each tissue was assumed to be species-independent and was kept the same as values derived from mouse biodistribution data (Table S5) [16]. A simple allometric equation PS × (body weight ratio)^b^ was employed to scale up PS in each tissue to higher species, where b = 0.75 and 1 (indicated species-independent) were tested for model performance optimization [11, 24, 25]. MMAE $\:{\text{C}\text{L}}_{\text{i}\text{n}\text{t}}$ determined using rat, monkey, and human liver microsothe mes were 32.9 mL/min/kg (range 30.2–35.6 mL/min/kg), 33.6 mL/min/kg (28.1–39 mL/min/kg), and 13.7 mL/min/kg (10.1–17.2 mL/min/kg), respectively [20, 22, 23]. On the other hand, $\:{\text{C}\text{L}}_{\text{i}\text{n}\text{t}}$ were 16.4 mL/min/kg (12.5–20.3 mL/min/kg), 10.6 mL/min/kg (8.8–12.3 mL/min/kg), and 2.35 mL/min/kg (1.2–3.5 mL/min/kg) when determined using rat, monkey, and human hepatocytes, respectively [20, 22, 23]. Both values derived from liver microsomes and hepatocytes were tested during model simulations, and the value that better match the observed data was selected for use in the PBPK model.

For the mAb component, the association and dissociation rate constants for FcRn binding in rats, monkeys, and humans were also derived from the literature [15]. The values representing the fraction of FcRn-bound mAbs recycled to the vascular space and the pinocytosis rate per unit endosomal volume were held constant across species [15]. Regarding ADC $\:{\text{k}}_{\text{d}\text{e}\text{c}}$ [26–35] and degradation rate constant ($\:{\text{k}}_{\text{d}\text{e}\text{g}}$) [30, 36–39], these parameters were assumed to vary across species. The information about the exact values that can be assigned to these parameters in each species was limited. Therefore, $\:{\text{k}}_{\text{d}\text{e}\text{c}}$ was fine-tuned by optimizing the parameter to capture the observed DAR vs. time profile for each species, and $\:{\text{k}}_{\text{d}\text{e}\text{g}}$ was optimized based on corroborative literature to enhance model performance for ADC PK in each species (detailed in the next section).

### PBPK model optimization and validation

The validation and optimization of the translational PBPK model for ADCs in rats, monkeys, and humans were conducted in two phases, an a priori simulation followed by *a posteriori* simulation. In the a priori simulation, species-specific physiological and drug-related parameters obtained from the literature were integrated into the PBPK model. The $\:{\text{k}}_{\text{d}\text{e}\text{c}}$ and $\:{\text{k}}_{\text{d}\text{e}\text{g}}$used for simulation remained consistent with the values optimized in the mouse ADC PBPK model (Table S4). In the second step (*a posteriori*), our aim was to refine $\:{\text{k}}_{\text{d}\text{e}\text{c}}$ and $\:{\text{k}}_{\text{d}\text{e}\text{g}}$ for each species to improve the model prediction of plasma and tissue PK for different ADC analytes. Optimization of $\:{\text{k}}_{\text{d}\text{e}\text{c}}$ was achieved by refining Eqs. 2 and 3, enabling the model to better characterize the DAR versus time profiles derived from observed PK data for each species. Conjugation of payloads to the mAb may introduce additional clearance that is unique to ADCs and not applicable to naked mAbs [36]. By comparing PK profiles and PK parameters between the total mAb component of the ADC and its naked mAb backbone, we quantitatively assessed the additional clearance attributed to payload conjugation. This information was then employed to optimize the $\:{\text{k}}_{\text{d}\text{e}\text{g}}$ parameter for each species. The concentrations of different ADC analytes in plasma and tissues predicted by the model, both in a priori and *a posteriori* simulations, were overlapped with observed data from the literature to assess the performance of the PBPK model. The simulations were conducted using Berkeley Madonna (Version 10, University of California at Berkeley, CA).

To evaluate model performance, the percent prediction error (%PE) for the area under the curve from time zero to the last observed time (AUC_0 − t_) was calculated by comparing observed and predicted PK profiles in each species using Eq. 4. AUC_0 − t_ of observed data were calculated by pooling all the literature-reported data together and applying the sparse sampling method in Phoenix WinNonlin (Version 8.4; Certara USA, Inc., 2023). The two-fold overprediction and underprediction correspond to %PE of 100% and − 100%, respectively.

4 $$\% {\rm{PE}} = \left\{ {\matrix{{\left( {1 - {{{\rm{AUC}}_{0 - {\rm{t}}}^{{\rm{Observed}}}} \over {{\rm{AUC}}_{0 - {\rm{t}}}^{{\rm{Predicted}}}}}} \right) \times {\kern 1pt} 100,} \hfill \cr {\,\,\,\,\,\,\,\,\,\,\,\,{\rm{AUC}}_{0 - {\rm{t}}}^{{\rm{Predicted}}} < {\rm{AUC}}_{0 - {\rm{t}}}^{{\rm{Observed}}}} \hfill \cr {\left( {{{{\rm{AUC}}_{0 - {\rm{t}}}^{{\rm{Predicted}}}} \over {{\rm{AUC}}_{0 - {\rm{t}}}^{{\rm{Observed}}}}} - 1} \right) \times \,100,} \hfill \cr {\,\,\,\,\,\,\,\,\,\,\,{\rm{AUC}}_{0 - {\rm{t}}}^{{\rm{Predicted}}} \ge \,{\rm{AUC}}_{0 - {\rm{t}}}^{{\rm{Observed}}}} \hfill \cr } } \right.$$

## Results

### Data collection

#### Data for rats

**Fig. 2:**
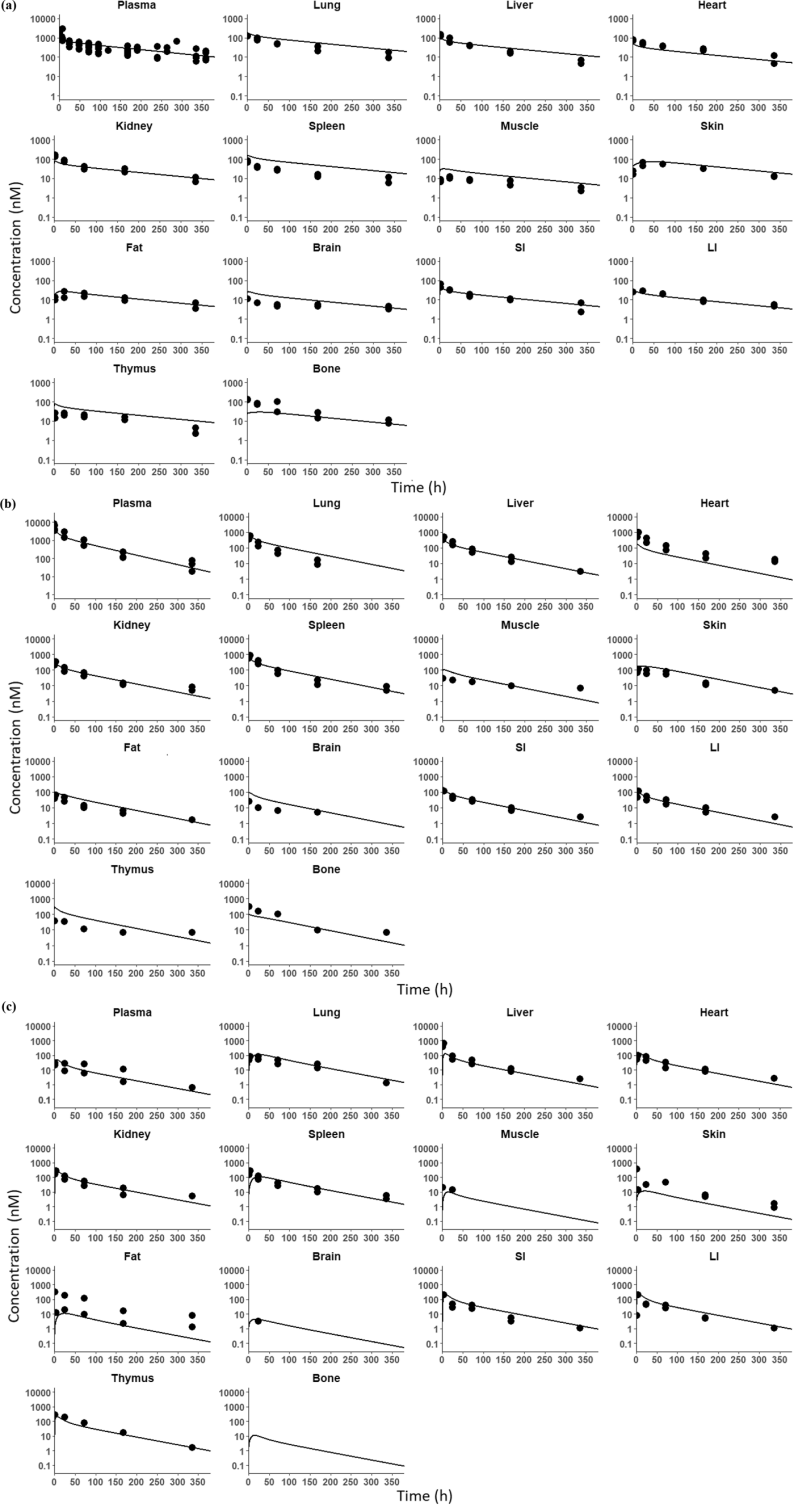
Comparison between literature-reported observed data and PBPK model-predicted plasma and tissue PK of MMAE-based ADCs in rats. The figures present the observed concentrations (circles) for (**a**) total antibody, (**b**) conjugated MMAE, and (**c**) unconjugated MMAE of ADCs, superimposed with *a posteriori* predictions from the PBPK model after optimization of deconjugation related parameter

A total of 8 references reporting PK of MMAE-based ADCs in rats were collected [13, 40–45]. Among these studies, two of the studies reported both plasma and tissues PK of different ADC analytes, and the other reported plasma PK of different ADC analytes [13, 40]. Yip et al. reported PK data in plasma and 19 tissues for polatuzumab vedotin using [^125^I]- and [^111^In]-mAb-labeled ADC as well as soluble and precipitable [^3^H]-MMAE-labeled ADC, which enabled retrieving whole-body disposition of total mAb, conjugated MMAE, and unconjugated MMAE for polatuzumab vedotin [40]. In Li et al. poster, PK profiles of an MMAE-based ADC were reported in plasma and 8 tissues for total mAb, conjugated MMAE, and unconjugated MMAE [13]. The other 6 references reported PK of total mAb and/or conjugated mAb of MMAE-based ADC in plasma [41–45]. All the collected concentrations of different ADC analytes in rats were dose-normalized to 10 mg/kg (Fig. 2).

#### Data for monkeys

**Fig. 3:**
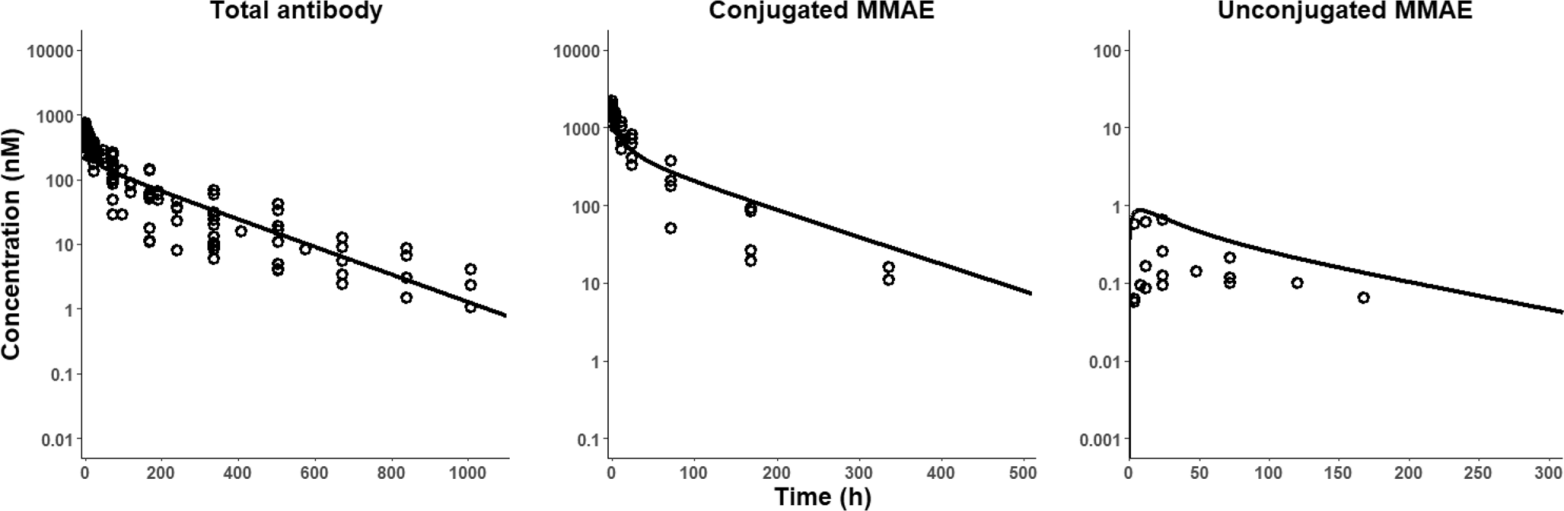
Comparison between literature-reported observed data and PBPK model-predicted plasma PK of MMAE-based ADCs in monkeys. The figure shows the observed concentrations (circles) for (**a**) total antibody, (**b**) conjugated MMAE, and (**c**) unconjugated MMAE of ADCs, superimposed with *a posteriori* predictions from the PBPK model after optimization of deconjugation related parameter

A total of 7 studies that reported PK of MMAE-based ADCs in monkeys were collected [42, 43, 45–49]. All the studies reported plasma PK profiles, while no studies reported tissue PK profiles. The reported ADC analytes included total mAb, conjugated mAb, conjugated MMAE, and unconjugated MMAE. All the collected concentrations of different ADC analytes in monkeys were dose-normalized to 5 mg/kg, which was the most commonly used dose in PK studies conducted in monkeys (Fig. 3).

#### Data for humans

**Fig. 4:**
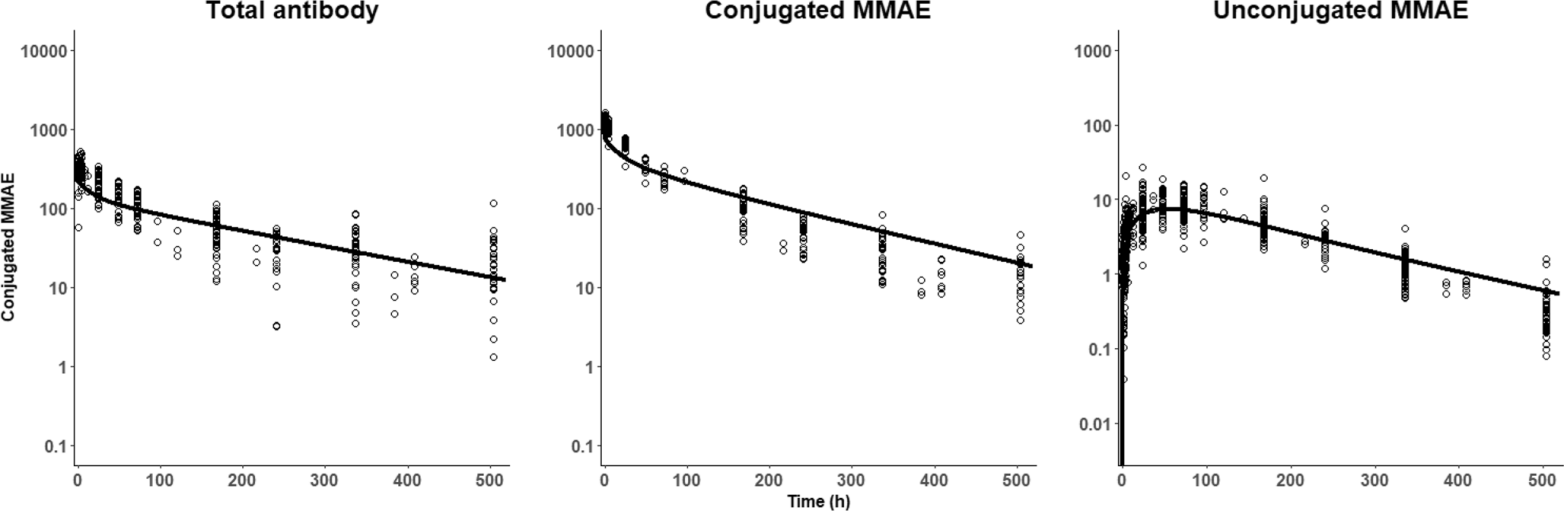
Comparison between literature-reported observed data and PBPK model-predicted plasma PK of MMAE-based ADCs in humans. The figure shows the observed concentrations (circles) for (**a**) total antibody, (**b**) conjugated MMAE, and (**c**) unconjugated MMAE of ADCs, superimposed with *a posteriori* predictions from the PBPK model after optimization of deconjugation related parameter

We collected clinical pharmacokinetic data for 18 distinct MMAE-based ADCs, resulting in a total of 109 mean PK profiles [18, 22, 23, 46, 50–79]. Five of these ADCs, including brentuximab vedotin, polatuzumab vedotin, tisotumab vedotin, enfortumab vedotin, and disitamab vedotin have been approved by the FDA. These profiles included data for total mAb, conjugated mAb, conjugated MMAE, and unconjugated MMAE. Previous works have demonstrated the generalizability of PK profiles across different MMAE-based ADCs [19], regardless of their targets, tumor indications, or average DAR, using dose-normalization techniques [18]. Therefore, all human PK data were dose-normalized to 2.4 mg/kg and subsequently pooled to develop a translational human PBPK model for ADCs (Fig. 4).

#### PBPK model simulation and optimization

Figures S1A-S1C show the a priori simulated PK profiles for total mAb, total MMAE, and unconjugated MMAE in rats, superimposed with observed data in plasma and 19 tissues. Figures S2 and S3 present a priori simulated plasma PK profiles for the three ADC analytes in monkeys and humans, respectively, overlapped with corresponding observed data. The simulation results suggest that the $\:{\text{C}\text{L}}_{\text{i}\text{n}\text{t}}$ values obtained from rat hepatocytes, monkey liver microsomes, and human hepatocytes lead to more accurate model predictions. Additionally, of the two reported MMAE protein bindings in monkeys, 18% offered better model predictions compared to 65% (data not shown), and thus, 18% was incorporated into the final PBPK model.

In rats, the model captured the plasma and tissue disposition of all three ADC analytes reasonably well, while at the last time point, model tend to slightly overpredict the concentrations of conjugated and unconjugated payloads in plasma and some tissues, indicating room for optimization of the $\:{\text{k}}_{\text{d}\text{e}\text{c}}$ parameter (Figure S1). In contrast, the a priori simulations showed systemic deviations from observed PK profiles in both monkeys and humans (Figure S2 and S3). Specifically, in monkeys, the model underpredicted the clearance of total mAb and tend to overpredict the observed concentration of unconjugated MMAE, failing to capture the time to peak concentration ($\:{\text{T}}_{\text{m}\text{a}\text{x}}$). In humans, the model underpredicted the clearance of total mAb while overpredicted the clearance of both conjugated and unconjugated payloads. Additionally, the use of the same $\:{\text{k}}_{\text{d}\text{e}\text{c}}$ across different species led to deviations between the predicted and observed DAR over time profiles in monkeys and humans. These findings suggested that the translational PBPK model requires further refinement in higher species and humans

**Fig. 5:**
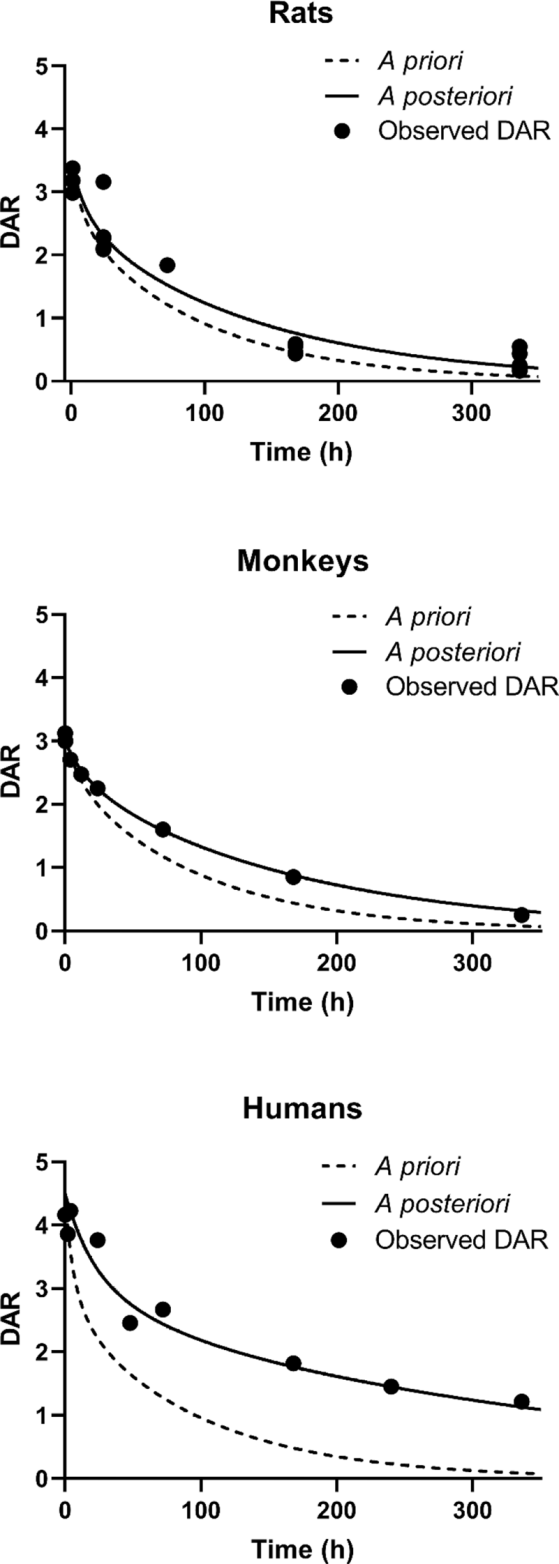
Comparison between observed and model-predicted DAR vs. time profiles in rats (upper), monkeys (middle), and humans (bottom). The figure displays the DAR vs. time profiles derived from literature-reported observed data (circles), superimposed on a priori (dashed line) and *a posteriori* (solid line) predictions of DAR vs. time profiles. The empirical equation k_dec_ = (α - ((α - β) × DAR^(-τ)) / (F + DAR^(-τ))) × scaling factor (SF) was used to describe the biphasic decline in the DAR vs. time profile, with α, β, F, and τ set at 0.0806, 0.0102, 0.282, and 5.68, respectively. The SF was calculated using a simple allometric scaling equation with an exponent of -0.25, resulting in SF values of 0.6 for rats, 0.3 for monkeys, and 0.1 for humans, respectively

To optimize deconjugation parameters, we initially investigated DAR over time profiles for collected ADCs in rats, monkeys, and humans (Fig. 5). A biphasic decline was observed in DAR over time profiles for all species, similar to what was observed in mice. Consequently, empirical equations used for $\:{\text{k}}_{\text{d}\text{e}\text{c}}$ that were optimized for describing this biphasic decline in mice were presumed to be applicable across species. However, considering that enzymatic activity may differ between species, thereby influencing relative deconjugation rates, we incorporated a scaling factor (SF) into Eq. 3. Utilizing a simple allometric scaling equation with an exponent of -0.25 enabled the model to capture DAR over time profiles across all species reasonably well (Fig. 5). The calculated SF were 0.6 for rats, 0.3 for monkeys, and 0.1 for humans, indicating that $\:{\text{k}}_{\text{d}\text{e}\text{c}}$ in these species were approximately 60%, 30%, and 10%, respectively, when compared to that in mice.

By comparing the PK profiles of the total mAb component of ADCs with the PK of naked unconjugated mAbs in mice [80], rats [81, 82], monkeys [83], and humans [84] (Figure S4), we found that the impact of conjugation-induced clearance varied among species. In mice and rats, the PK profiles of the total mAb component of ADCs (i.e., trastuzumab-vc-MMAE) were relatively comparable to those of naked mAbs (i.e., trastuzumab). This suggested that MMAE conjugation did not significantly alter the systemic PK of the mAb component in rodents. In contrast, for monkeys and humans, the total mAb component of MMAE-based ADCs exhibited enhanced clearance compared to their naked mAb backbones, corresponding to previously reported PK parameters. Moreover, the average clearance for the total mAb of ADCs was approximately 16 mL/day/kg in both monkeys (range ~ 14–22 mL/day/kg) and humans (~ 7–28 mL/day/kg). In comparison, data from multiple approved mAb therapeutics indicated that the average clearance for naked mAbs was around 6 mL/day/kg (~ 3–12 mL/day/kg) in monkeys and 4 mL/day/kg (~ 2–6 mL/day/kg) in humans. These findings suggested that MMAE conjugation could increase the clearance by approximately three- and four-fold in monkeys and humans, respectively, when compared to the naked mAb backbones. Based on this analysis, we assumed that $\:{\text{k}}_{\text{d}\text{e}\text{g}}$ in rats was consistent with the value optimized for naked mAbs, while $\:{\text{k}}_{\text{d}\text{e}\text{g}}$ in monkeys and humans were set at three- and four-fold higher, respectively, than the optimized $\:{\text{k}}_{\text{d}\text{e}\text{g}}$ for naked mAbs.

Upon incorporating the optimized $\:{\text{k}}_{\text{d}\text{e}\text{c}}$ and $\:{\text{k}}_{\text{d}\text{e}\text{g}}$ values, *a posteriori* predictions for ADC pharmacokinetics were performed. Figure 2a and b, and 2c display the plasma and tissue disposition of total mAb, conjugated MMAE, and unconjugated MMAE in rats, which closely aligned with the observed data. The PBPK model was able to predict plasma and tissue PK profiles for all these analytes in rats reasonably well. The %PE for total mAb, conjugated MMAE, and unconjugated MMAE in rats is detailed in Table S6 for plasma and Table S7 for individual tissues. The prediction of tissue PK for total mAb in rats were within a 2-fold range, with the exception of spleen concentrations, which were slightly overpredicted but remained within a 3-fold range. The predictions of tissue PK for conjugated payload were within a 2-fold range, with an exception of slight underpredictions for bone and heart (within a 3-fold range) and slight overpredictions for thymus. The predictions of PK for unconjugated payload in tissues were within a 2-fold range for most tissues, while the model underpredicted concentrations in fat and skin. However, the variability in reported observed fat concentrations of unconjugated payload might contribute to the higher %PE in this tissue.

Figures 3 and 4 present *a posteriori* predictions for plasma PK profiles of the three ADC analytes in monkeys and humans, overlapped with literature-collected data. The %PE for plasma PK of total mAb, conjugated MMAE, and unconjugated MMAE in monkeys and humans are provided in Table S6. The model characterized the PK of total mAb (%PE -6%) and conjugated MMAE (%PE 3%) in plasma well, while it overpredicted the plasma PK of unconjugated MMAE; however, the prediction was within a 3-fold range. The ADC PBPK model accurately characterize the plasma PK profiles for total mAb (%PE -0.718%), conjugated MMAE (%PE -4.45%), and unconjugated MMAE (%PE -12.6%) of ADCs in humans simultaneously.

#### Application of ADC PBPK model in clinical scenarios

To evaluate the clinical relevance of our model in predicting efficacy, we integrated the ADC PBPK model with a tumor model published previously [85] and incorporated clinically relevant parameters to simulate the concentration of free payload within tumors in patients. Since our previous PBPK model for ADCs was developed using HER2-expressing tumor-bearing mice, we extended the model to simulate payload exposure in human tumors with varying levels of HER2 expression. HER2 concentration levels for IHC1+, IHC2+, IHC3+, and triple-negative breast cancer (TNBC) were approximated based on existing literature [86–88].

For our simulations, we assumed a tumor size of 2.5 cm in diameter (T2 category) and utilized PK parameters from a previous tumor model developed for HER2-positive tumors. Simulations were conducted for both single-dose and multiple-dose regimens following currently approved dosing schedules (i.e., every 3 weeks or weekly for 3 doses within a 28-day cycle). Our results demonstrated that higher target expression levels led to increased tumor payload exposure (Figures S5a-c), corroborating our previous findings [89]. These findings suggest that payload exposure data may offer insights into a reliable exposure-efficacy relationship for MMAE-based ADCs in clinical settings.

## Discussion

ADCs utilize mAbs to selectively deliver cytotoxic agents to cancer cells. However, the advantage of targeted delivery comes at the expense of more complex PK, as it is affected by the combined effect of the mAb backbone, payload, and linker. While measuring plasma concentrations of ADC analytes is relatively straightforward, assessing tissue exposure poses a significant challenge in clinical settings. A thorough understanding of tissue PK is essential for establishing reliable exposure-toxicity relationships for ADCs. To address this, we employed a PBPK modeling framework that facilitates efficient cross-species translation of both model structure and parameters. Building upon our previously developed dual-structured whole-body PBPK model for mice [11], we expanded this model to encompass higher species and humans. Our translational PBPK model was validated using plasma and tissue disposition data sourced from the literature for multiple ADCs and species. The translational PBPK model was able to simultaneously capture tissue exposure of different ADC analytes, thereby facilitating the identification of PK determinants that drive ADC-related toxicities observed in clinical trials going forward.

To predict drug PK in humans, allometric scaling methods have widely been employed, and many studies have proven to successfully predict the PK of mAb therapeutics through allometric scale-up of clearance and volume of distribution. However, the straightforward application of allometric scaling may not suit ADCs, due to their complexity, influenced by factors such as molecular size, linker type, and drug loading. In contrast, our study employs a PBPK modeling framework, supported by literature data and allometric principles, which provides a platform for scaling PK of ADC between species. While previous studies have developed PBPK models for MMAE-based ADCs using in vitro, preclinical, and clinical data [5, 6, 8], these models often lacked full dual PBPK framework, thereby limiting their ability to simultaneously characterize tissue disposition for both the mAb and payload components of an ADC. Our study addresses these limitations by establishing a translational PBPK model that mechanistically describes the complexities of ADC disposition, offering a platform for PK scale-up. This approach enables the simultaneous prediction of multiple ADC analytes in individual tissues. Additionally, the translational PBPK model structure is flexible, as it can be updated to characterize the PK of ADCs with novel designs (i.e., different linker-payload) once data are available.

The development of the translational PBPK model for ADCs involved several steps. First, we gathered biodistribution data for MMAE-based ADCs in multiple species. Dose-normalized PK profiles for individual ADCs within the same species were comparable, indicating the absence of notable target binding effects on ADC PK. This finding corroborates previous studies suggesting that the PK of individual ADCs was generalizable, irrespective of their respective targets and indications [19]. Accordingly, we pooled the PK data from individual ADCs within the same species for model validation. Second, information regarding MMAE disposition was gathered from in vitro and in vivo data reported in the literature. PK parameters describing mAb disposition for each species were extracted from our previously published platform PBPK model for mAbs. Third, the mouse PBPK model was scaled up to rats, monkeys, and humans by incorporating species-specific drug-related and physiological parameters. This was followed by a priori simulations for the PK of different ADC analytes in each species, superimposed with observed data. Lastly, PK parameters associated with payload release processes were optimized in each species based on supportive evidence from the literature. *A posteriori* simulations employing these optimized PK parameters were overlapped with observed data to evaluate model performance.

PK parameters describing MMAE disposition were sourced from various publications, and we noted relative variations in reported values for several key parameters, including monkey MMAE $\:{\text{f}}_{\text{u}\text{p}}$ and $\:{\text{C}\text{L}}_{\text{i}\text{n}\text{t}}$ in individual species. Our strategy involved incorporating each reported value of these parameters into the PBPK model for predictive analysis, ultimately selecting those values that best aligned the predicted PK profiles with observed data. Specifically, MMAE $\:{\text{f}}_{\text{u}\text{p}}$ of 18% in monkeys provided more accurate model predictions than 65% (data not shown). Additionally, the literature reported a wide range of $\:{\text{C}\text{L}}_{\text{i}\text{n}\text{t}}$ for each species, with values derived from liver microsomes generally higher than those obtained via hepatocytes. We therefore included the mean values of $\:{\text{C}\text{L}}_{\text{i}\text{n}\text{t}}$ from either source in our model simulations and evaluated performance across species. Our results indicated that the $\:{\text{C}\text{L}}_{\text{i}\text{n}\text{t}}$ obtained from rat hepatocytes, monkey liver microsomes, and human hepatocytes led to more accurate model predictions. There are some additional considerations regarding the elimination of MMAE in the PBPK model. First, in the MMAE PBPK model, CL_int_ was assigned to the interstitial space to effectively capture hepatic metabolism, given the estimated PS value, liver blood flow rate, and the assumption of rapid exchange between plasma and the interstitial compartment. While a more detailed model of hepatic metabolism could further refine the PBPK framework, the current methodology remains a physiologically reasonable representation of MMAE clearance given the available data. Second, we did not explicitly incorporate biliary excretion of unconjugated MMAE due to limited quantitative data in humans and across preclinical species and recognize that this omission may lead to an underestimation of total hepatic clearance. However, given that the reported intrinsic clearance values from in vitro hepatocyte and microsome studies exhibit up to a three-fold variability, a potential two-fold difference resulting from the exclusion of biliary excretion remains within the range of reported data.

The allometric scaling principle was examined for parameters including PS and $\:{\text{K}}_{\text{d}\text{e}\text{c}}$ in the PBPK model. Initially, we compared PS values in each tissue scaled up using a simple allometric approach to those kept constant across species. We found that allometric scaling of PS did not enhance model predictions; thus, PS values in each tissue were kept the same across all species. For $\:{\text{K}}_{\text{d}\text{e}\text{c}}$, the SF assigned to the equation was scaled up using a simple allometric equation. The scaling up of SF with an exponent of -0.25 allowed for accurate predictions of the DAR versus time profiles across multiple species. This $\:{\text{K}}_{\text{d}\text{e}\text{c}}$ optimization suggests higher rates of deconjugation for MMAE-based ADCs in lower species. Our findings could be explained by species-specific expression and tissue distribution of the enzyme carboxylesterases 1c, a primary contributor to the extracellular payload release of ADCs and their instability in vivo [36]. Carboxylesterases 1c is highly active and expressed in mouse plasma, less so in rat plasma, and not expressed in monkey and human plasma. Moreover, the tissue distribution of carboxylesterases 1c varies among species (i.e., carboxylesterases 1c is expressed in the small intestine and kidney in monkeys but not in humans) [27, 29, 30, 33, 34]. Applying simple allometric scaling to $\:{\text{K}}_{\text{d}\text{e}\text{c}}$ enabled the model to reasonably capture both the conjugated and released payload across different species.

It has been reported that payload conjugation may induce additional ADC clearance [36]. However, there is limited data to directly support the applicability of this additional clearance across species and its quantitative impact on ADC clearance in each species. To assess the influence of conjugation on ADC clearance, we compared PK profiles and parameters between naked mAbs and the total mAb component of ADCs in different species. Our findings suggest that the extent of conjugate-induced clearance varies across species, with significant effects observed in higher species and in clinical settings, but not in rodents. This may be explained by the fact that differences in the immune cell population across species may contribute to species-specific immune cell uptake and degradation [39]. It has been reported that the interaction of ADCs with Fcγ receptors on immune cells can lead to enhanced clearance [90, 91]. Our PBPK modeling indicates that k_deg_ is approximately 3–4-fold greater in monkeys and humans, which is quantitatively supported by the approximately 3–5-fold higher abundance of FcγR-bearing cells (i.e., monocytes, macrophages, and neutrophils) in these species compared to rodents [92]. Additionally, since our comparison focused on the total mAb component of ADCs rather than the conjugated mAb, differences in clearance in higher species are more likely attributed to variations in degradation rather than deconjugation, as the latter would not affect the PK of the total mAb component of ADCs. In our model, we employed species-specific degradation rates ($\:{\text{K}}_{\text{d}\text{e}\text{g}}$) to account for the observed differences in conjugate-induced clearance of ADC across species. However, additional parameters like $\:{\text{C}\text{L}}_{\text{u}\text{p}}$ and additional processes could be optimized/included to further characterize this phenomenon once more data become available.

The ADC PBPK model in humans, integrated with a tumor model and incorporating clinically relevant parameters, facilitates understanding of efficacy across different dosing regimens and disease states, such as HER2 expression levels. Accordingly, we simulated receptor occupancy dynamics across various dosing regimens and IHC expression levels to assess their impact on tumor payload exposure. Our findings suggest that receptor occupancy dynamics play a key role in tumor payload PK particularly in the context of varying antigen expression levels and dosing regimens. Our simulations of receptor occupancy profiles across different IHC expression levels (Figure S6) indicate that while IHC3 + tumors exhibit lower receptor occupancy than IHC2 + tumors, their higher total antigen burden results in greater overall ADC uptake and sustained intracellular MMAE release. This is reflected in the similar tumor payload concentrations observed between IHC2 + and IHC3 + tumors under Q3W dosing regimen, as well as the higher MMAE exposure in IHC3 + tumors under the weekly dosing regimen. Furthermore, more frequent dosing provides a more stable and sustained MMAE release in tumors compared to Q3W dosing, reducing fluctuations in payload exposure. Therefore, increasing dosing frequency may improve therapeutic efficacy by maintaining tumor exposure above the IC50 threshold. However, this approach also elevates systemic exposure to free MMAE, potentially increasing the risk of toxicity.

After developing the translational PBPK model, it can be further applied to investigate key questions related to the safety of ADCs. The translational PBPK model is capable of predicting the distribution of various ADC analytes in tissues, as shown in Figure S7, thereby facilitating the investigation of ADC safety profiles in clinics. This predictive capability for tissue PK could assist in identifying which exposure matrices and ADC analytes drive the observed toxicities associated with MMAE-based ADCs in clinical settings. However, due to the lack of clinical biopsy data, these findings require further validation. If tumor biopsy-derived PK measurements become available in clinical settings, these data could be used to refine PBPK models, particularly the tumor compartment, to improve their translational accuracy. Additionally, we utilized the model for pathway analysis to evaluate the relative contributions of individual tissues, tumors, and the deconjugation process to the generation of unconjugated payload from ADCs in plasma (Figure S8). The results align with previous studies [11], which suggest that while the deconjugation process from ADCs significantly contributes to the initial generation of unconjugated drug in plasma, the contribution from tissues rapidly becomes the predominant source for sustained exposure. Furthermore, the contribution of tumors to unconjugated payload exposure in plasma was found to be lower than many normal tissues. This suggests that tumor burden may not demonstrate strong correlation with plasma PK of certain ADC analytes and proves the fact that despite the targeting ability and stability of ADCs, the free payload is generated all over the body. In fact, the model predicted relatively high released payload concentrations in healthy tissues such as the liver, spleen, and kidneys, which suggests potential for ADC-associated toxicities in these tissues that could lead to the narrow therapeutic index.

While the translational PBPK model generally predicts the whole-body distribution of various ADC analytes across species, there are some limitations to consider. Specifically, the prediction of payload components (i.e., conjugated or unconjugated MMAE) in certain rat tissues, as well as the prediction of unconjugated MMAE plasma PK in monkeys, were slightly imprecise. This may be attributed to the assumption of constant single Kp values, which can actually be concentration-dependent [93, 94]. Furthermore, the current PBPK model assumes that Kp values for individual tissues remain constant across species. To further evaluate this, we conducted a sensitivity analysis by varying Kp in each tissue and evaluating its impact on unconjugated MMAE AUC across rats, monkeys, and humans (Figure S9). The results show that the effect of increasing or decreasing Kp by 20% on unconjugated MMAE AUC follows a similar pattern across species. High-perfusion tissues (i.e., lung, heart, kidney) were more sensitive to Kp changes, indicating that a constant Kp assumption may introduce higher variability for these tissues. Future PBPK model refinements could incorporate concentration-dependent Kp, particularly for high-perfusion tissues, to improve tissue-level accuracy. Due to the limited availability of tissue data, the model’s predictions for tissues in higher species (i.e., monkeys and humans) could not be validated. Therefore, further validation of tissue PK for payload exposure using imaging studies or other quantitative approaches is encouraged, as accurate prediction of tissue payload exposure is critical for evaluating toxicity, particularly for drugs with a narrow therapeutic window, such as ADCs. Nonetheless, the proposed model still serves as a platform to develop reliable exposure-response relationships for ADCs going forward (See Table 1).

**Table 1:** Species-specific Pharmacokinetic parameters used in the translational PBPK model for MMAE based adcs

Parameters (unit)	Definition	Mice	Rats	Monkeys	Humans
Protein binding (%)^a^	MMAE plasma protein binding	20.0	87.5	18.0^b^	76.7
$\:{\text{K}}_{\text{P},\text{B}\text{C}}$ ^c^	MMAE red blood cell partition	5.46	4.00	3.00	1.34
$\:{\text{C}\text{L}}_{\text{i}\text{n}\text{t}}$ (mL/min/kg)^c, d^	MMAE intrinsic clearance	67.7	16.4	33.6	2.35
$\:{\text{k}}_{\text{o}\text{n}}^{\text{F}\text{c}\text{R}\text{n}}$ (1/nM/h)^d^	Association rate constant between mAb and FcRn	0.0806	0.800	0.792	0.559
$\:{\text{k}}_{\text{o}\text{f}\text{f}}^{\text{F}\text{c}\text{R}\text{n}}$ (1/h)^e^	Dissociation rate constant between mAb and FcRn	6.55	144	46.8	23.9
$\:\text{f}\_{\text{K}}_{\text{d}\text{e}\text{g}}$ ^f^	Optimized fold-change of degradation rate for ADC	1	1	3	4
$\:\text{S}\text{F}$ ^g^	Optimized scaling factor for ADC deconjugation rate	1	0.6	0.3	0.1

Weights used in the model are 28 g for mice, 280 g for rats, 6.2 kg for monkeys, and 70 kg for humans

^a^ Values obtained from [11, 14, 23, 27, 28]

^b^ Two values of MMAE protein binding in monkeys, 18% and 65%, were reported, and the 18% was chosen for the final model because of its better model prediction

^c^ Values for mice were obtained from [11], and values for rats, monkeys, and humans were obtained from [14, 23, 27, 28]

^d^ For rats, monkeys, and humans, mean values within the reported ranges were used. $\:{\text{C}\text{L}}_{\text{i}\text{n}\text{t}}$ values, derived from rat hepatocytes, monkey liver microsomes, and human hepatocytes, provided better model predictions and were thus used in the final model

^e^ Values obtained from [4]

^f^ For rats, monkeys, and humans, $\:{\text{K}}_{\text{d}\text{e}\text{g}}$ values were derived by multiplying $\:\text{f}\_{\text{K}}_{\text{d}\text{e}\text{g}}$ with the mouse $\:{\text{K}}_{\text{d}\text{e}\text{g}}$

^g^ The scaling factor (SF) for rats, monkeys, and humans were calculated using the simple allometric scaling method with an exponent of -0.25, SF = (weight/mouse weight)^−0.25^. The deconjugation rate constant was determined by multiplying SF with the $\:{\text{K}}_{\text{d}\text{e}\text{c}}$ equation (see Eq. 2)

We acknowledge that some mechanisms influencing ADC tissue distribution were not included in the current PBPK model, such as target expression and P-gp transport in tissues. Specifically, target-mediated drug disposition (TMDD) in tissues was not incorporated, as the dataset we collected contained limited data from cross-reactive ADCs in rats and the lacked PK data across a wide dose range (Table 2), preventing an evaluation of its impact on ADC disposition. Future refinements incorporating TMDD-driven tissue disposition could improve tissue PK predictions [95]. Additionally, MMAE is a known substrate of P-gp, and further incorporating P-gp efflux transport in tissues such as the liver and intestine could enhance the utility of the ADC PBPK model in assessing drug-drug interactions (DDIs) related to the released payload of ADCs [96].

**Table 2:** Literature-reported PK data for different analytes of MMAE-Based ADCs across various species

Species	References (year) or ADC name	ADC targets	ADC analytes	Dose (mg/kg)
Rats^a^	Yip et al. (2021) [41]	CD79b	Plasma: Total mAb, unconjugated MMAE Tissues: Total mAb, unconjugated/conjugated MMAE	10
	Li. et al. (2017) [13]	CD79b	Plasma: Total mAb, unconjugated/conjugated MMAE Tissues: Unconjugated/conjugated MMAE	5
	Boswell et al. (2011) [42]	STEAP1	ADC, total mAb	5
	Lin et al. (2015) [43]	NaPi2B	Total mAb	0.5, 5
	Bhakta et al. (2017) [44]	GDNF	Total mAb	0.5, 5
	Chuprakov et al. (2021) [45]	CD79b	ADC, total mAb	20
	Zhang et al. (2021) [46]	DR5	ADC, total mAb	10
Monkeys^a^	Polivy EMA (2019) [47]	CD79b	Total mAb, conjugated MMAE	0.3, 1, 3
	Li et al. (2019) [48]	CD79b	Total mAb	1, 3, 5
	Lu et al. (2014) [49]	CD79b	Total mAb, unconjugated/conjugated MMAE	0.3, 1, 3
	Lin et al. (2015) [43]	NaPi2B	Total mAb	0.3, 1.0
	Bhakta et al. (2017) [44]	GFRA1	Total mAb	0.3, 3
	Zhang et al. (2021) [46]	DR5	ADC, total mAb, unconjugated MMAE	4.0
	Sanderson et al. (2005) [50]	CD30	Total mAb	0.3, 3.0
Humans	Brentuximab vedotin	CD30	ADC, unconjugated MMAE	1.2–2.7
	Polatuzumab vedotin	CD79b	Total mAb, unconjugated/conjugated MMAE	0.1–2.4
	Pinatuzumab vedotin	CD22	Total mAb, unconjugated/conjugated MMAE	2.4
	Lifastuzumab vedotin	Napi2b	Total mAb, unconjugated/conjugated MMAE	2.4
	Losatuxizumab vedotin	EGFR	ADC, total mAb, unconjugated MMAE	0.3-3.0
	Tisotumab vedotin	Tissue factor	ADC, unconjugated MMAE	0.3–2.2
	Telisotuzumab vedotin	c-Met	ADC, total mAb, unconjugated MMAE	0.15–3.3
	Glembatumumab vedotin	gpNMB	ADC, total mAb, unconjugated MMAE	1.88
	ASG-5ME	SLC44A4	ADC, total mAb, unconjugated MMAE	0.3-3.0
	Ladiratuzumab vedotin	LIV-1	ADC, unconjugated MMAE	1.0-2.5
	PSMA ADC	PSMA	ADC, unconjugated MMAE	0.4–2.8
	Vandortuzumab vedotin	Steap1	Total mAb, conjugated MMAE	0.33–2.4
	DEDN6526A	ETBR	Total mAb, unconjugated/conjugated MMAE	2.4
	DMOT4039A	MsLN	Total mAb, unconjugated/conjugated MMAE	2.4
	DMUC5754A	MUC16	Total mAb, unconjugated/conjugated MMAE	2.4
	DFRF4539A	FcRH5	Total mAb, unconjugated/conjugated MMAE	2.4
	Enfortumab vedotin	Nectin-4	ADC, total mAb, unconjugated MMAE	0.5–1.25
	Disitamab vedotin	HER2	ADC, total mAb, unconjugated MMAE	0.1-3.0

^a^For rats, ADCs in References [13, 40–42, 44] were cross-reactive, while ADCs in References [43, 45] were not cross-reactive. For monkeys, all ADCs tested in the studies were cross-reactive

## Conclusion

In this study, a previously developed whole-body PBPK model for MMAE-based ADCs is scaled to higher preclinical species and humans. Species-specific physiological and drug-related parameters for the payload and the mAb backbone were sourced from the literature and integrated into the PBPK model. Parameters related to payload release were further optimized for each species. Kdec was fine-tuned using the allometric scaling approach, while Kdeg was adjusted to account for conjugate-enhanced clearance of ADCs in each species. Based on these optimized parameters, we observed a lower deconjugation rate and enhanced in vivo stability of ADCs in higher species. On the other hand, the impact of payload conjugation on ADC clearance tends to be more pronounced in higher species and humans. The translational PBPK model presented here can facilitate the prediction of PK profiles for various ADC analytes at the site-of-action. This information could inform exposure-toxicity and exposure-efficacy relationships, and may potentially help with the optimization of the therapeutic window for ADCs.

## Electronic supplementary material

Below is the link to the electronic supplementary material.

Supplementary Material 1

## Data Availability

No datasets were generated or analysed during the current study.
