# Supplementary material for: A translational physiologically-based pharmacokinetic model for MMAE-based antibody-drug conjugates
Source: J Pharmacokinet Pharmacodyn. 2025 May 5;52(3):27. doi: 10.1007/s10928-025-09978-3 (PMC12053227; doi:10.1007/s10928-025-09978-3)
Supplement: Supplementary file 1 — Supplementary Material 1 [file 10928_2025_9978_MOESM1_ESM.docx]

**Electronic Supplementary Material**

**A Translational Physiologically-Based Pharmacokinetic Model for MMAE-Based Antibody-Drug Conjugates**

Hsuan-Ping Chang^a^ and Dhaval K. Shah^a,*^

*^a^Department of Pharmaceutical Sciences, School of Pharmacy and Pharmaceutical Sciences, The State University of New York at Buffalo, Buffalo, NY*

*Corresponding author:

Dhaval K. Shah, PhD

Department of Pharmaceutical Sciences

455 Pharmacy Building, School of Pharmacy and Pharmaceutical Sciences

University at Buffalo, The State University of New York

Buffalo, New York 14214-8033

Telephone: 716-645-4819

E-mail: [dshah4@buffalo.edu](mailto:dshah4@buffalo.edu)

**Figure legends**

**Fig. S1**. Comparison between literature-reported observed data and PBPK model-predicted plasma and tissue disposition of MMAE-based ADCs in rats. The figures present the observed concentrations (circles) for (a) total antibody, (b) conjugated MMAE, and (c) unconjugated MMAE of ADCs, superimposed with *a priori* predictions from the PBPK model without optimization of the deconjugation pathway.

**Fig. S2** Comparison between literature-reported observed data and PBPK model-predicted plasma PK of MMAE-based ADCs in monkeys. The figure shows the observed concentrations (circles) for (a) total antibody, (b) conjugated MMAE, and (c) unconjugated MMAE of ADCs, superimposed with *a priori* predictions from the PBPK model without optimization of the deconjugation and degradation pathway.

**Fig. S3** Comparison between literature-reported observed data and PBPK model-predicted plasma PK of MMAE-based ADCs in humans. The figure shows the observed concentrations (circles) for (a) total antibody, (b) conjugated MMAE, and (c) unconjugated MMAE of ADCs, superimposed with *a priori* predictions from the PBPK model without optimization of the deconjugation and degradation pathway.

**Fig. S4** Comparison between PK profiles of total mAb of MMAE-based ADCs (red circles) and naked mAbs (blue circles) in mice, rats, monkeys, and humans. Data for total mAb of MMAE-based ADCs are listed in Table 1, and data for naked mAbs were sourced from [60] for mice, [61, 62] for rats, [63] for monkeys, and [64] for humans.

**Fig. S5**. Model predicted clinical PK of unconjugated MMAE in tumors with different levels of HER2 expression following administration of MMAE-based ADCs at: (a) single dose, (b) every three weeks for 6 doses, and (c) Days 1, 8, and 15 of a 28-day cycle for 6 cycles.

**Fig. S6**. Model predicted receptor occupancy profiles over time for IHC1+, IHC2+, and IHC3+ tumors under (a) 1.8 mg/kg every 3 weeks and (b) 1.8 mg/kg weekly dosing regimen. The antigen levels used for simulation are 14.4 nM, 82.7 nM, and 650 nM for IHC1+, IHC2+, and IHC3+, respectively.

**Fig. S7.** Model-predicted clinical PK of unconjugated MMAE in tissues following MMAE-based ADC administration in humans for every three weeks for 5 doses.

**Fig. S8**. Pathway analysis assessing the relative importance of different tissues, tumors, and the deconjugation process on the disposition of MMAE in plasma after: (a) a single dose, and (b) every three weeks for 6 doses.

**Fig. S9**. Sensitivity analysis of partition coefficient (Kp) assumptions on unconjugated MMAE exposure in tissues across species. The percentage change in unconjugated MMAE AUC in each tissue was evaluated after increasing (white bars) or decreasing (black bars) Kp by 20% in (a) rats, (b) monkeys, and (c) humans, using the formula

$$\%Change= \frac{{AUC}_{original}-{AUC}_{\pm20\%}}{{AUC}_{original}}\times100$$

**Fig S1**

(a)


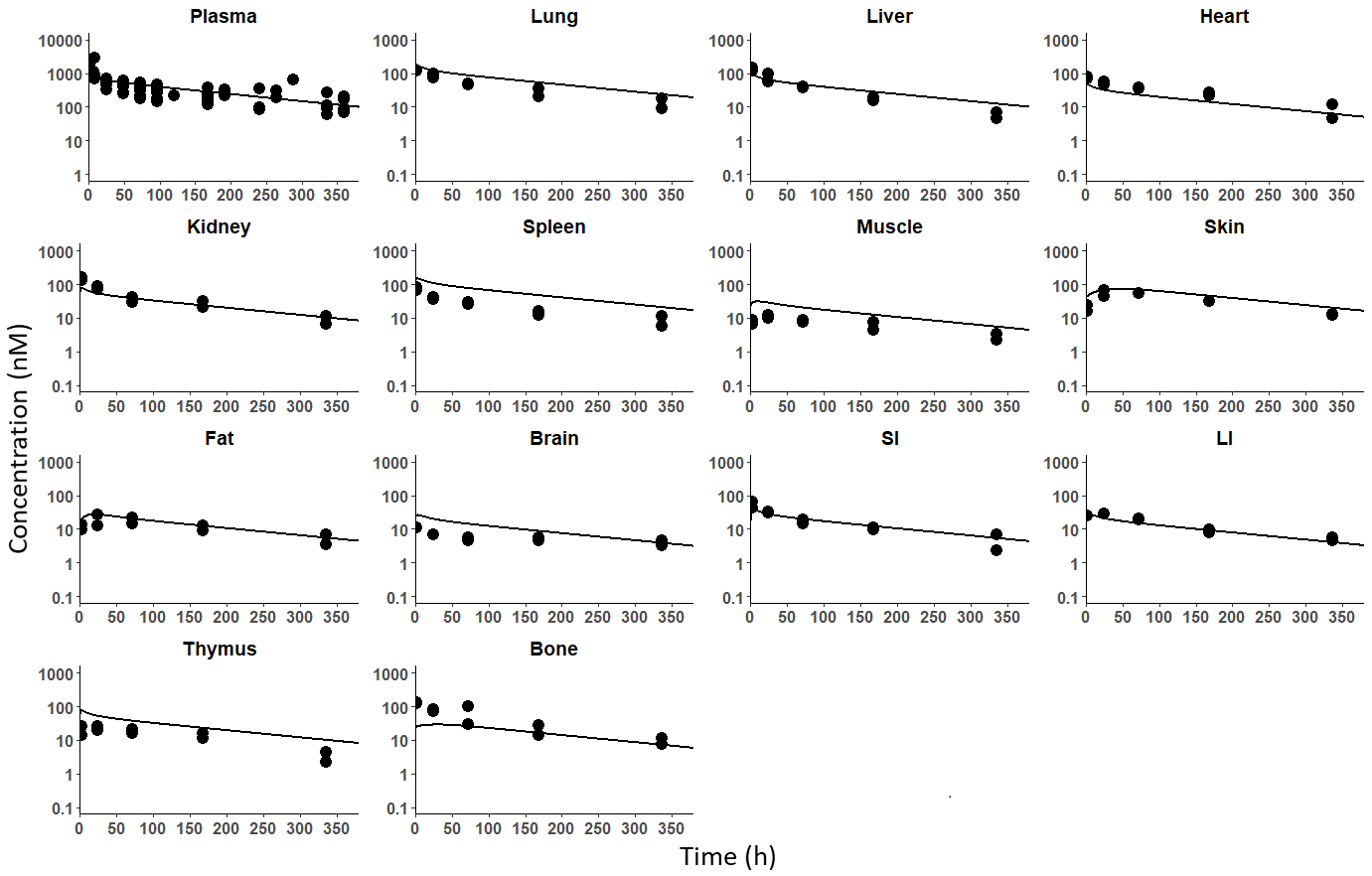


**Fig S1**

**(b)**

**
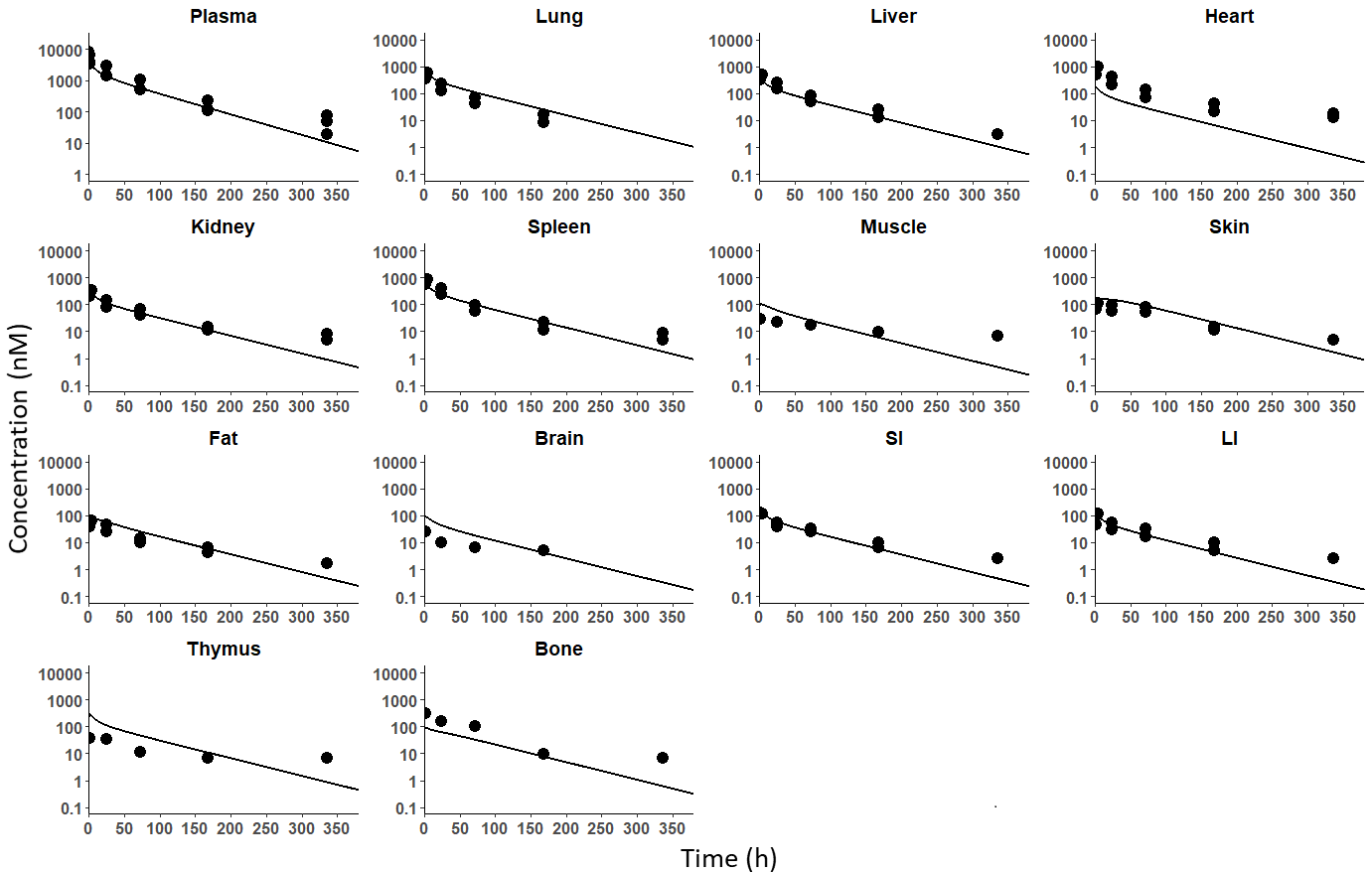
**

**Fig S1**

**(c)**

**
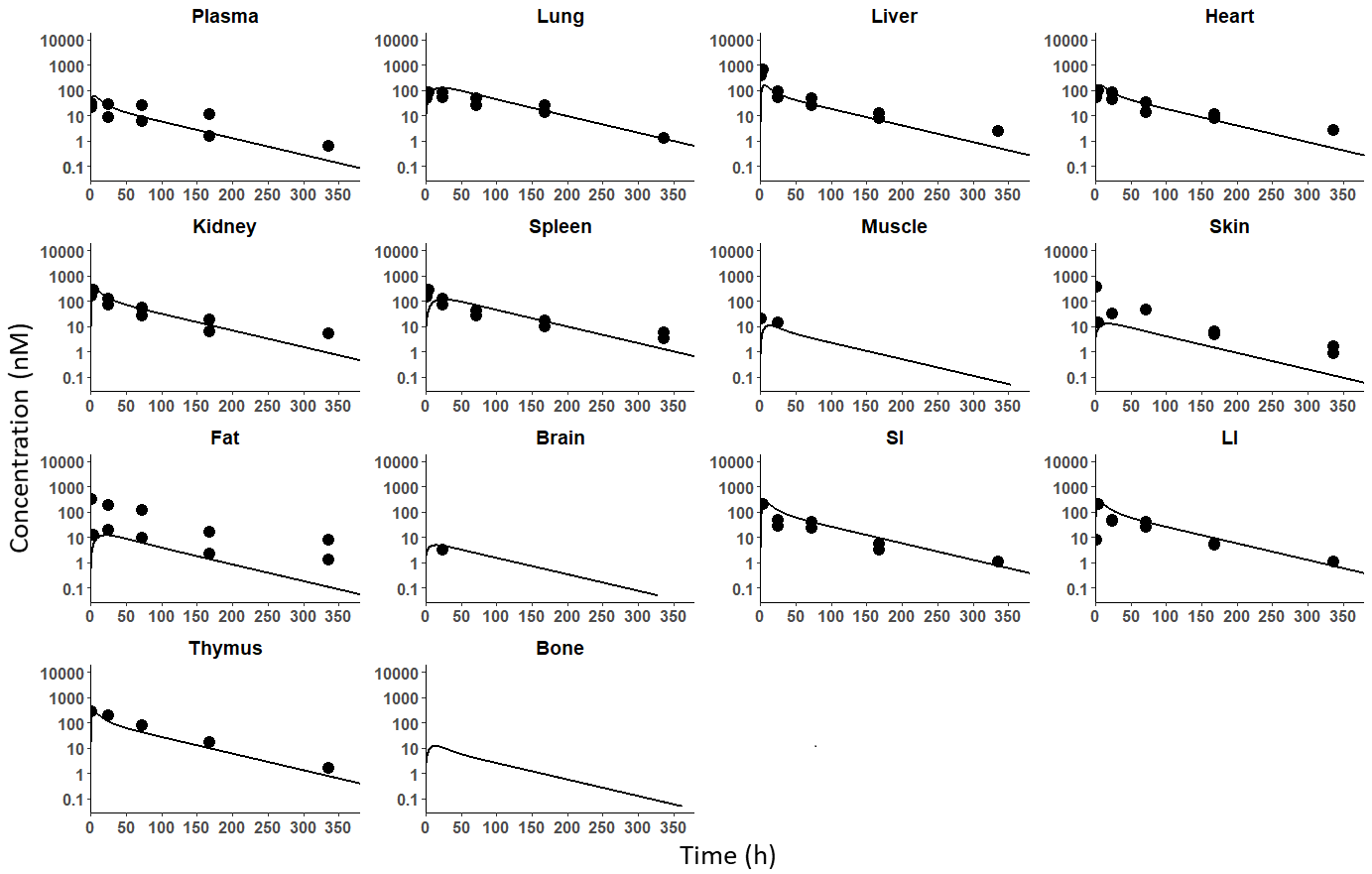
**

**Fig. S2**

**
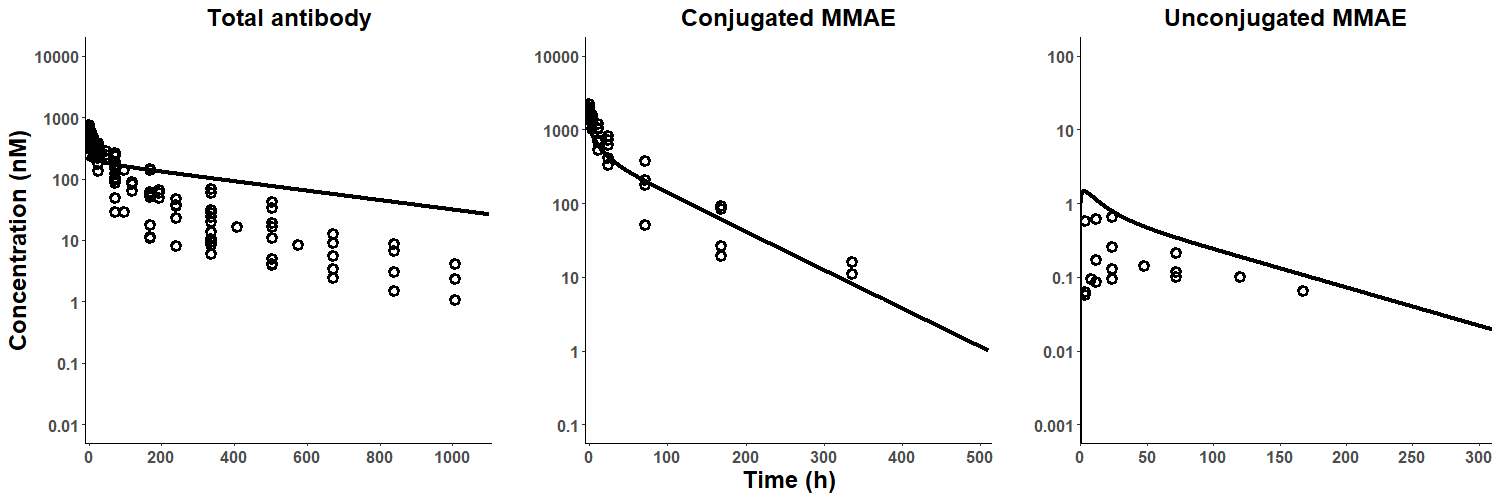
**

**Fig. S3**


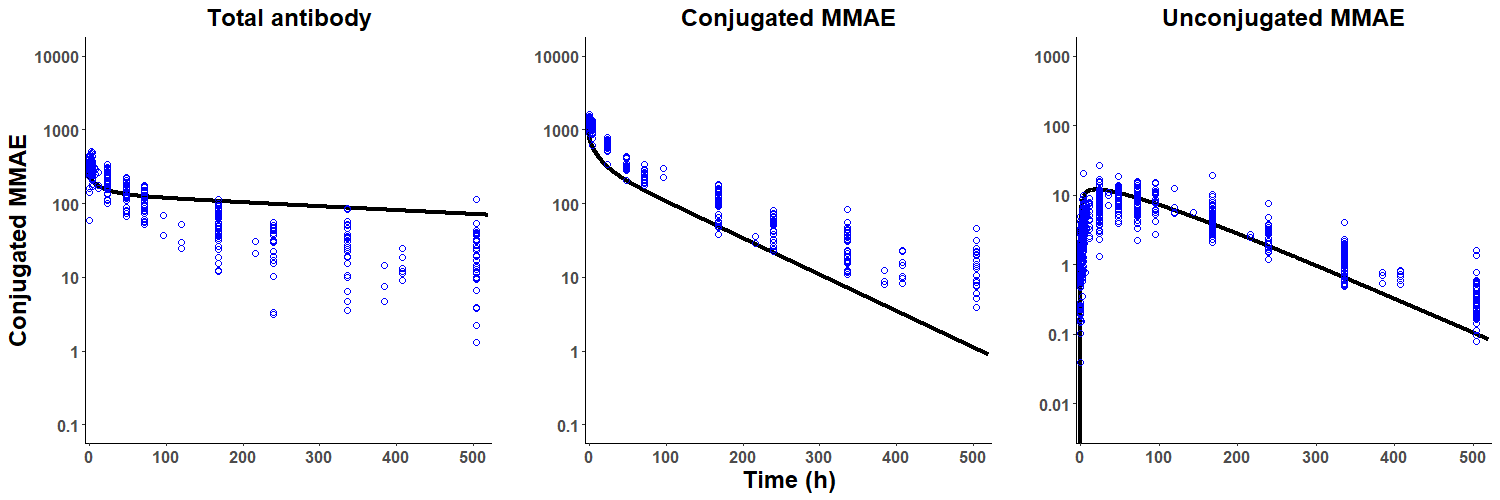


**Fig. S4**


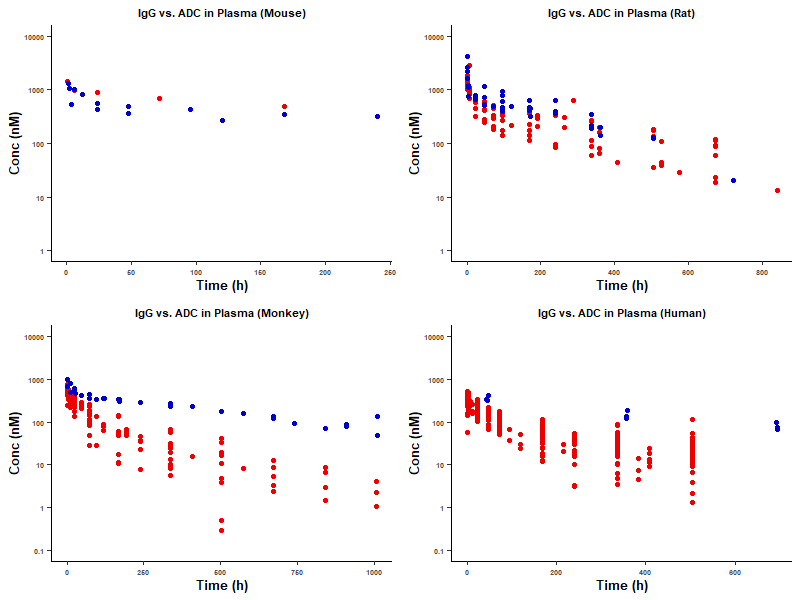


**Fig. S5**

(**a**)


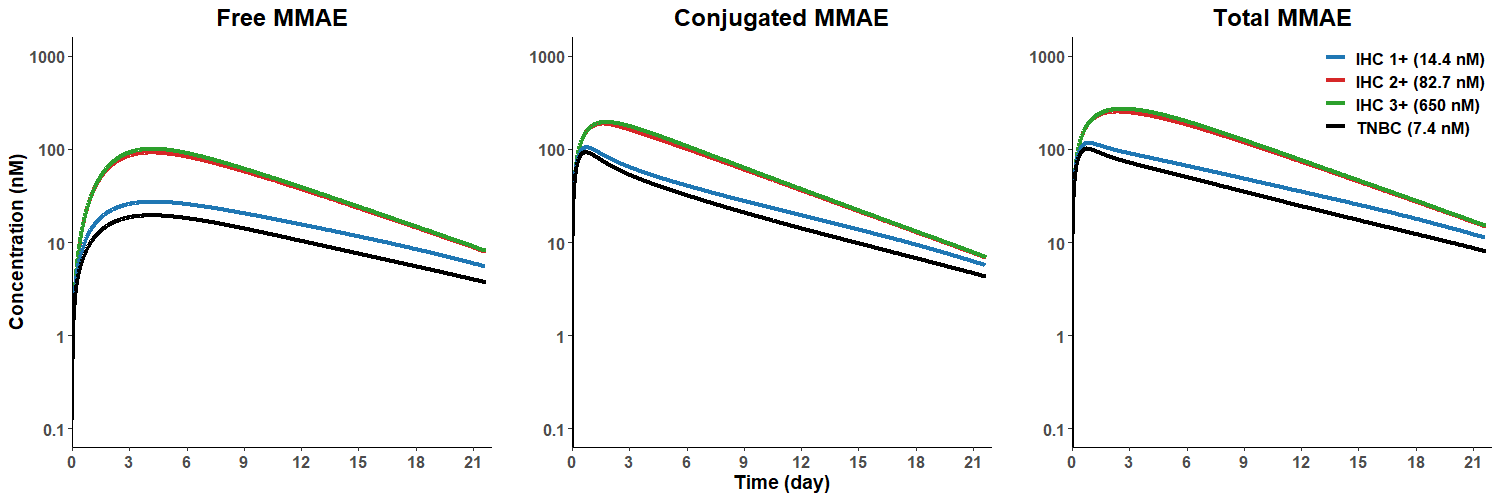


(**b**)


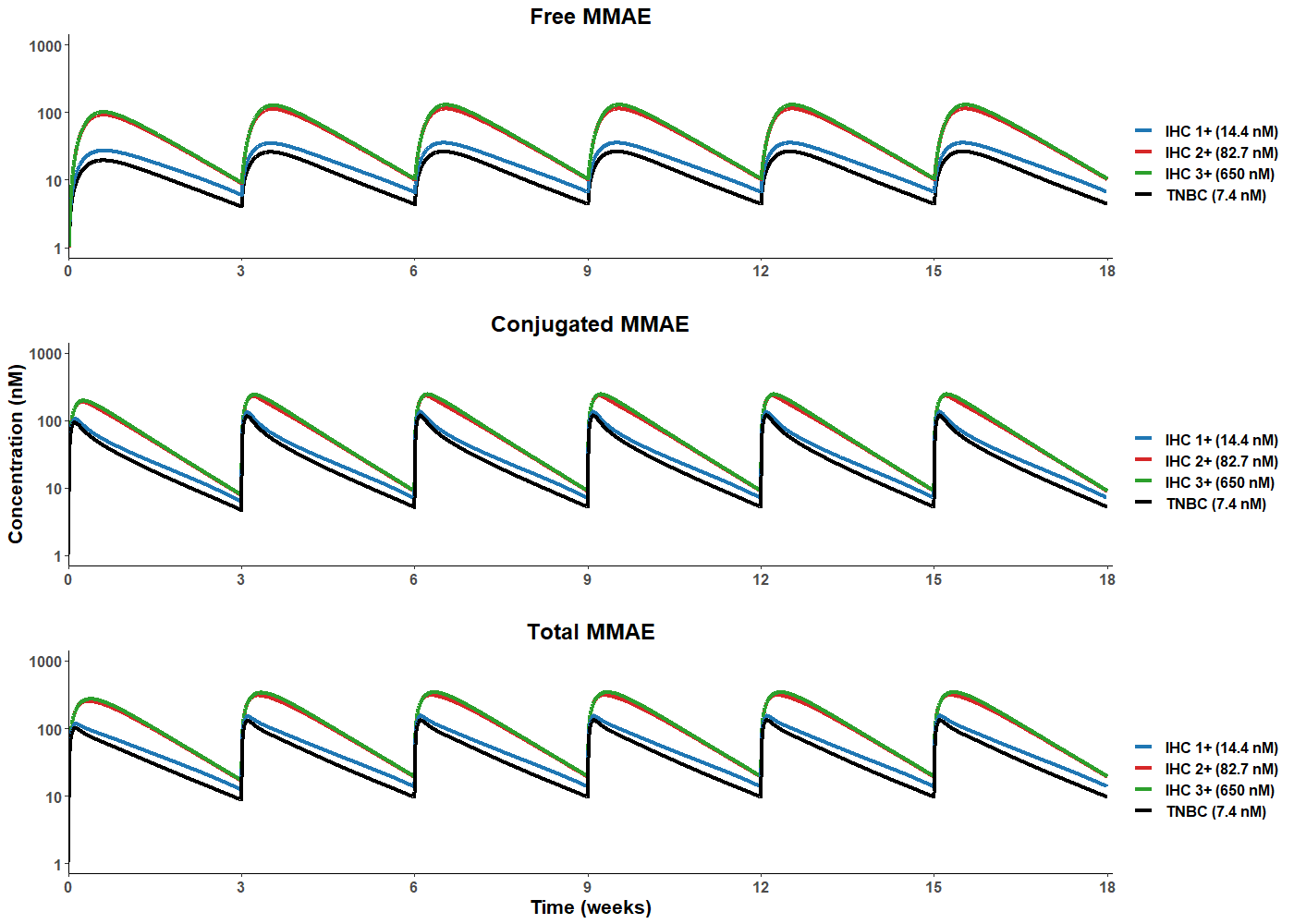


(**c**)


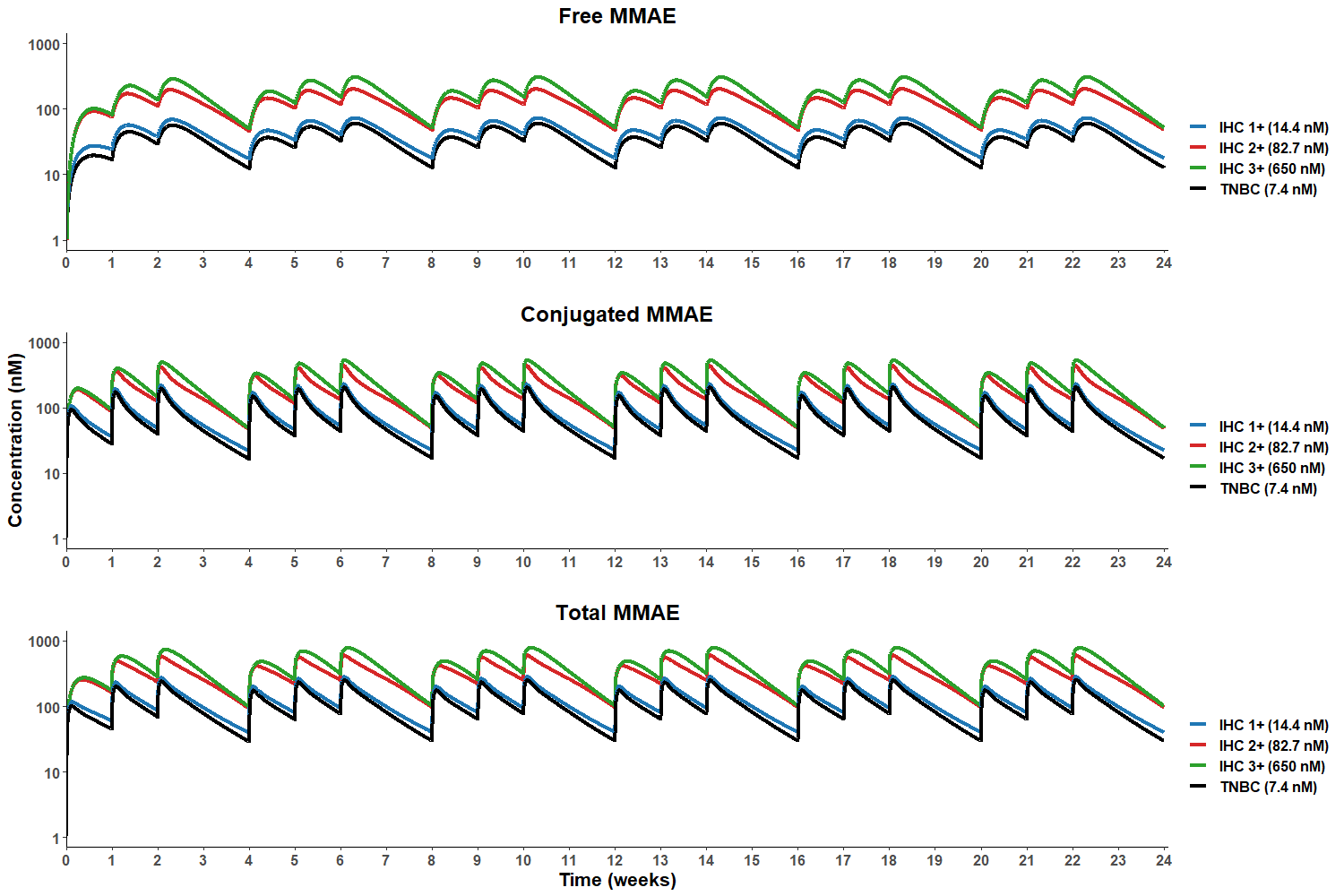


**Fig. S6**

(**a**)


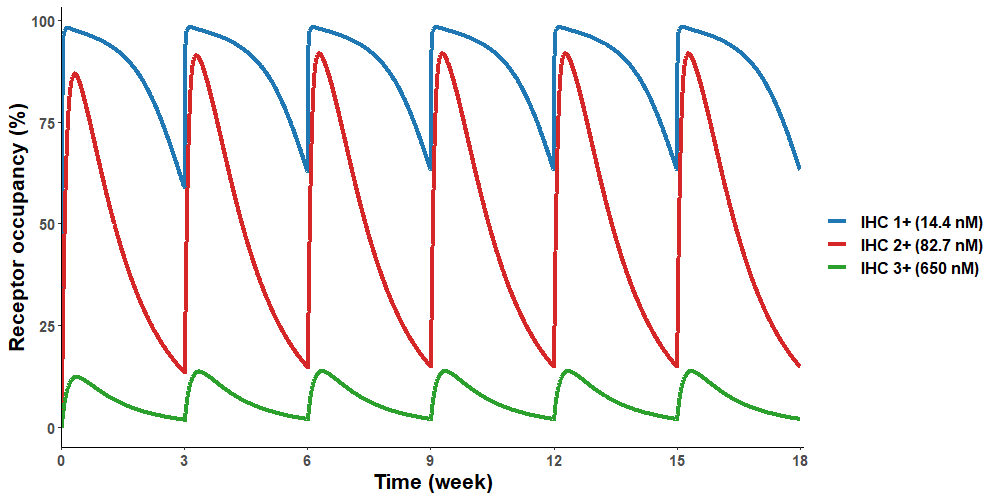


**(b)**


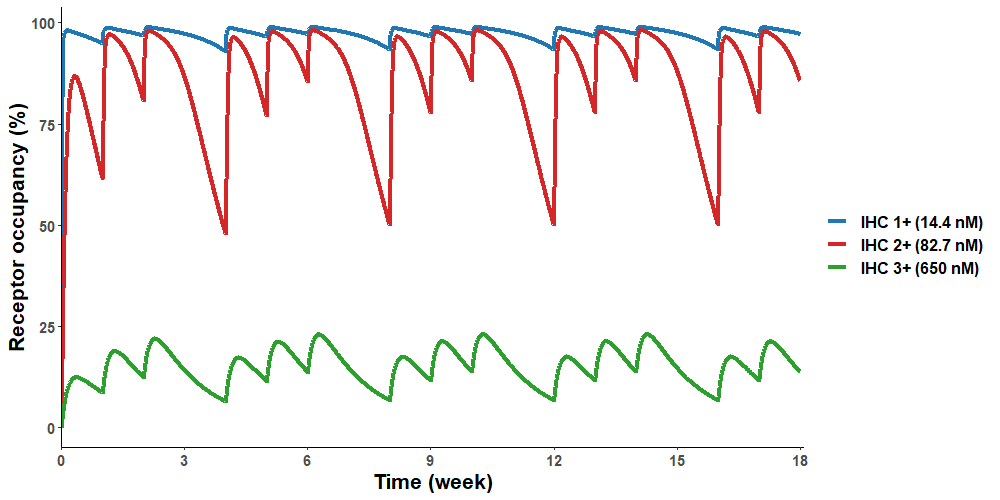


**Fig. S7**


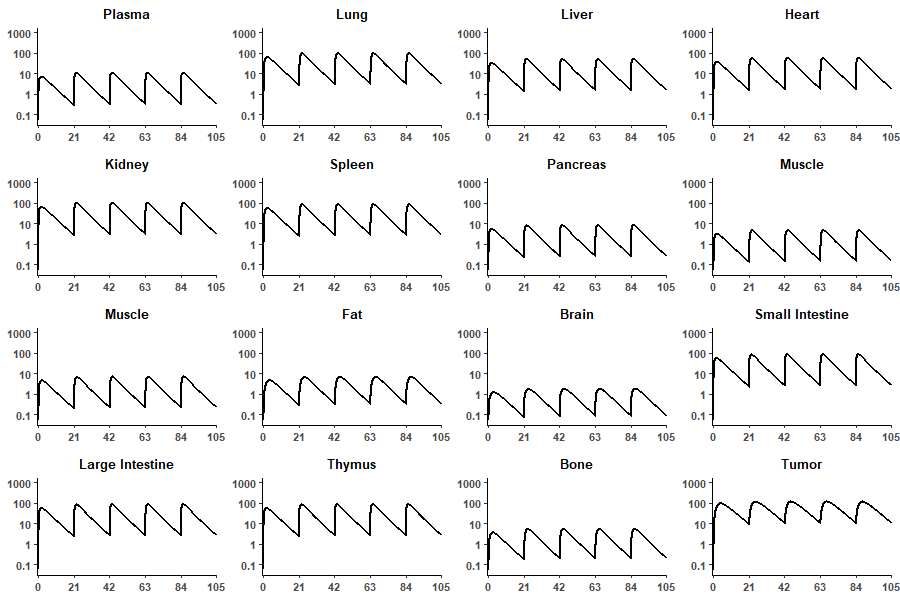


**Fig. S8**

(**a**)


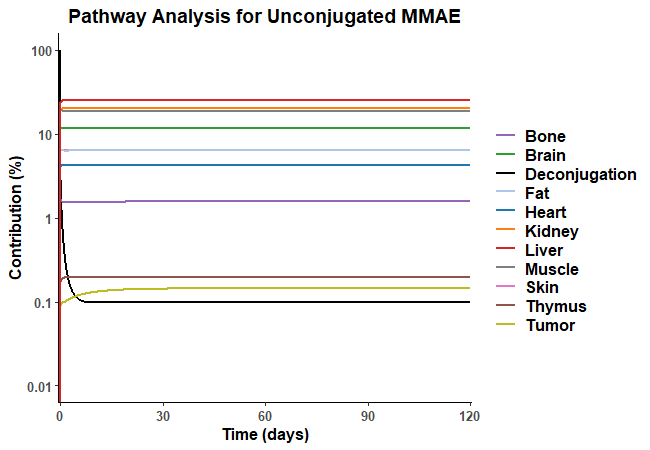


(**b**)


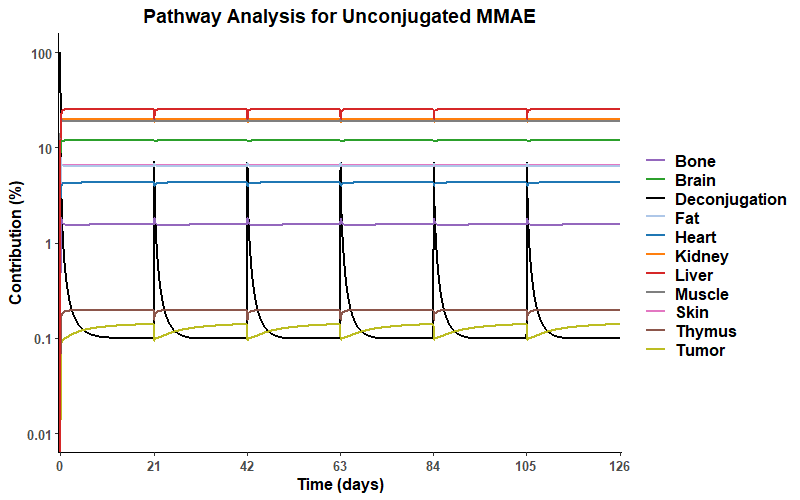


**Fig. 9**

(a) (b) (c)


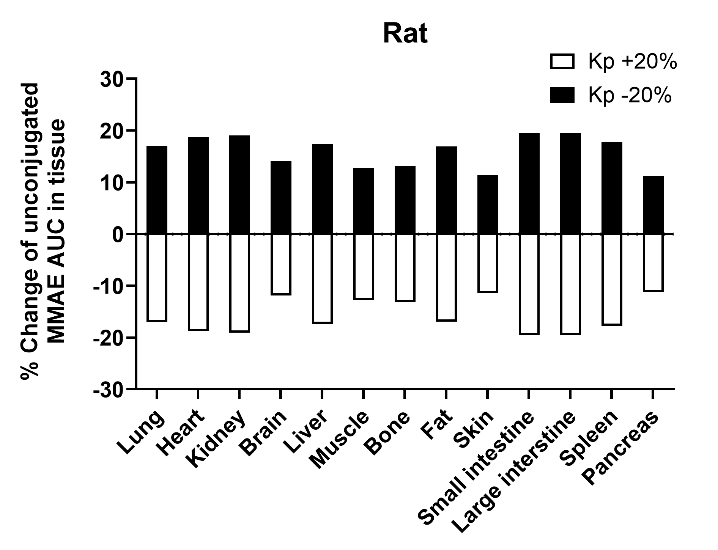

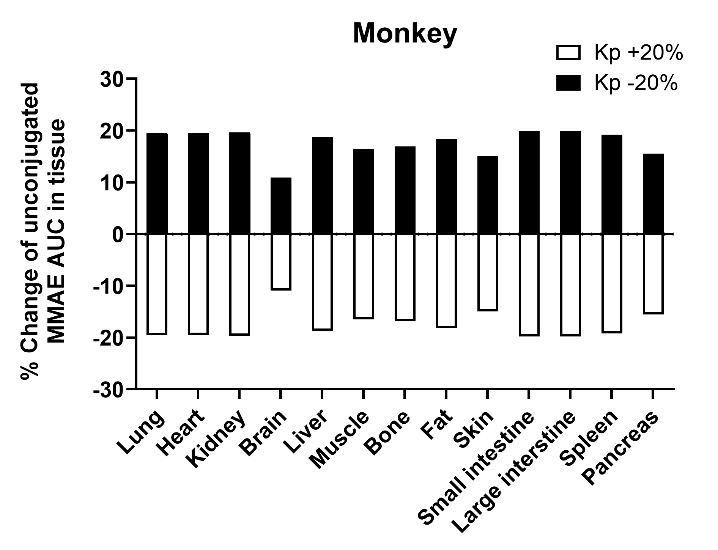

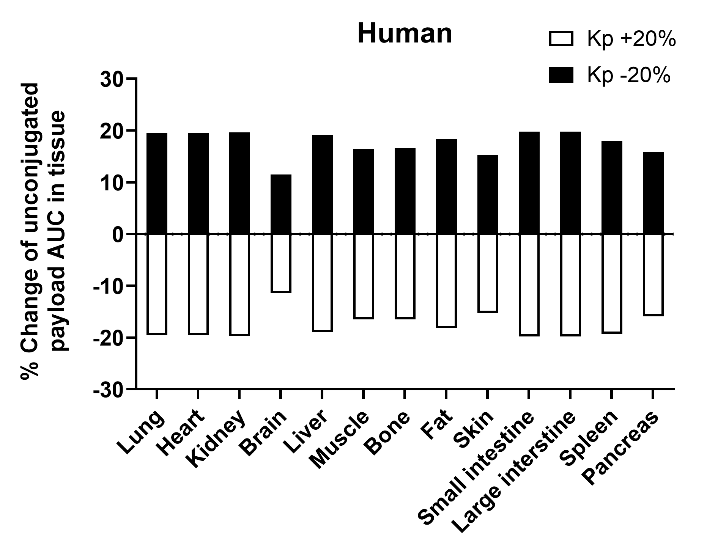


**Table S1** Physiological parameters used in the translational PBPK model for MMAE-based ADC in rats

|  | **Total volume (mL)** | **Plasma volume (mL)** | **Blood cell volume (ml)** | **Interstitial volume (mL)** | **Endosomal volume (mL)** | **Cellular volume (mL)** | **Plasma flow (mL/h)** | **Blood cell flow (mL/h)** |
| --- | --- | --- | --- | --- | --- | --- | --- | --- |
| Heart | 1.02 | 0.0394 | 0.0323 | 0.146 | 0.00512 | 0.801 | 151 | 124 |
| Lung | 1.40 | 0.231 | 0.189 | 0.263 | 0.00700 | 0.710 | 2945 | 2409 |
| Muscle | 122 | 2.68 | 2.19 | 15.8 | 0.608 | 100 | 925 | 757 |
| Skin | 49.9 | 1.87 | 1.53 | 16.5 | 0.249 | 29.8 | 200 | 163 |
| Adipose | 33.1 | 0.364 | 0.298 | 5.63 | 0.166 | 26.7 | 224 | 184 |
| Bone | 21.0 | 0.462 | 0.378 | 3.90 | 0.105 | 16.1 | 61.4 | 50.2 |
| Brain | 2.28 | 0.0502 | 0.0410 | 0.410 | 0.0114 | 1.77 | 65.3 | 53.5 |
| Kidney | 2.41 | 0.132 | 0.108 | 0.361 | 0.0120 | 1.79 | 365 | 298 |
| Liver | 15.7 | 1.34 | 1.10 | 2.56 | 0.0787 | 10.7 | 21.1 | 17.3 |
| Small Intestine | 4.99 | 0.0795 | 0.0651 | 0.867 | 0.0249 | 3.95 | 398 | 325 |
| Large Intestine | 2.87 | 0.0458 | 0.0375 | 0.500 | 0.0144 | 2.28 | 158 | 130 |
| Pancreas | 1.00 | 0.0547 | 0.0448 | 0.173 | 0.00498 | 0.717 | 63.4 | 51.8 |
| Thymus | 0.0960 | 0.00528 | 0.00432 | 0.0163 | 0.000480 | 0.0696 | 12.2 | 10.0 |
| Spleen | 2.77 | 0.335 | 0.274 | 0.554 | 0.0138 | 1.59 | 179 | 146 |
| Lymph Node | 1.15 | – | – | – | – | 1.15 | 16.8 | 13.8 |
| Other | 6.09 | 0.256 | 0.209 | 1.04 | 0.0305 | 4.55 | 105 | 85.6 |
| Plasma | 9.06 | – | – | – | – | – | 2945 | – |
| Blood cells | 7.41 | – | – | – | – | – | – | 2409 |

Parameters based on 280 g male rats

**Table S2** Physiological parameters used in translational PBPK model for MMAE-based ADC in monkeys

|  | **Total volume (mL)** | **Plasma volume (mL)** | **Blood cell volume (ml)** | **Interstitial volume (mL)** | **Endosomal volume (mL)** | **Cellular volume (mL)** | **Plasma flow (mL/h)** | **Blood cell flow (mL/h)** |
| --- | --- | --- | --- | --- | --- | --- | --- | --- |
| Heart | 28.3 | 1.09 | 0.892 | 4.05 | 0.142 | 22.2 | 696 | 570 |
| Lung | 35.7 | 1.96 | 1.61 | 10.7 | 0.179 | 21.2 | 22433 | 18355 |
| Muscle | 3273 | 72.0 | 58.9 | 426 | 16.4 | 2701 | 3944 | 3227 |
| Skin | 674 | 25.2 | 20.6 | 223 | 3.37 | 403 | 2492 | 2039 |
| Adipose | 154 | 1.70 | 1.39 | 26.3 | 0.772 | 124 | 139 | 113 |
| Bone | 952 | 20.9 | 17.1 | 177 | 4.76 | 732 | 248 | 203 |
| Brain | 94.0 | 2.07 | 1.69 | 16.9 | 0.470 | 72.9 | 1508 | 1234 |
| Kidney | 27.3 | 1.50 | 1.23 | 4.09 | 0.136 | 20.3 | 3237 | 2649 |
| Liver | 187 | 15.9 | 13.0 | 37.4 | 0.934 | 120 | 1251 | 1023 |
| Small Intestine | 98.7 | 1.57 | 1.29 | 17.2 | 0.494 | 78.2 | 3432 | 2808 |
| Large Intestine | 140 | 2.24 | 1.83 | 24.4 | 0.702 | 111 | 3571 | 2921 |
| Pancreas | 12.5 | 0.685 | 0.560 | 2.17 | 0.0623 | 8.98 | 399 | 327 |
| Thymus | 2.09 | 0.115 | 0.0941 | 0.355 | 0.0105 | 1.52 | 125 | 103 |
| Spleen | 5.95 | 0.720 | 0.589 | 1.19 | 0.0298 | 3.42 | 185 | 151 |
| Lymph Node | 25.1 | – | – | – | – | 25.1 | 363 | 297 |
| Other | 132 | 5.56 | 4.55 | 22.7 | 0.662 | 98.9 | 845 | 691 |
| Plasma | 187 | – | – | – | – | – | 22433 | – |
| Blood cells | 153 | - | - | - | - | - | - | 18355 |

Parameters based on 6.2 kg male monkeys

**Table S3** Physiological parameters used in translational PBPK model for MMAE-based ADC in humans

|  | **Total volume (mL)** | **Plasma volume (mL)** | **Blood cell volume (ml)** | **Interstitial volume (mL)** | **Endosomal volume (mL)** | **Cellular volume (mL)** | **Plasma flow (mL/h)** | **Blood cell flow (mL/h)** |
| --- | --- | --- | --- | --- | --- | --- | --- | --- |
| Heart | 341 | 13.1 | 10.8 | 48.8 | 1.71 | 267 | 7752 | 6342 |
| Lung | 1000 | 55.0 | 45.0 | 300 | 5.00 | 595 | 181913 | 148838 |
| Muscle | 30078 | 662 | 541 | 3910 | 150 | 24815 | 33469 | 27383 |
| Skin | 3408 | 127 | 104 | 1125 | 17.0 | 2035 | 11626 | 9512 |
| Adipose | 13465 | 148 | 121 | 2289 | 67.3 | 10840 | 11233 | 9191 |
| Bone | 10165 | 224 | 183 | 1891 | 50.8 | 7817 | 2591 | 2120 |
| Brain | 1450 | 31.9 | 26.1 | 261 | 7.25 | 1124 | 21453 | 17553 |
| Kidney | 332 | 18.2 | 14.9 | 49.8 | 1.66 | 247 | 36402 | 29784 |
| Liver | 2143 | 183 | 149 | 429 | 10.7 | 1371 | 13210 | 10808 |
| Small Intestine | 385 | 6.15 | 5.03 | 67.1 | 1.93 | 305 | 12368 | 10120 |
| Large Intestine | 548 | 8.74 | 7.15 | 95.3 | 2.74 | 434 | 12867 | 10527 |
| Pancreas | 104 | 5.70 | 4.66 | 18.0 | 0.518 | 74.7 | 3056 | 2500 |
| Thymus | 6.41 | 0.353 | 0.288 | 1.09 | 0.0321 | 4.65 | 353 | 289 |
| Spleen | 221 | 26.8 | 21.9 | 44.3 | 1.11 | 127 | 6343 | 5189 |
| Lymph Node | 274 | - | - | - | - | 274 | 3670 | 3002 |
| Other | 4852 | 204 | 167 | 831 | 24.3 | 3626 | 5521 | 4517 |
| Plasma | 3126 | - | - | - | - | - | 181913 | - |
| Blood cells | 2558 | - | - | - | - | - | - | 148838 |

Parameters based on 71 kg male humans

**Table S4** A glossary and literature-derived parameters used in translational PBPK model for MMAE-based ADCs.

| **Parameter** | **Definition** | **Value** | **Unit** |
| --- | --- | --- | --- |
| $Q_{plasma}^{i}$, $Q_{BC}^{i}$, $Q_{lymph}^{i}$ | Flow rate to the tissue “i” | - | L/h |
| $V_{plasma}^{i}$, $V_{BC}^{i}$, $V_{IS}^{i}$,$V_{endo}^{i}$, $V_{cellular}^{i}$ | Volume of vascular, blood cell, endosomal, interstitial, and cellular compartment for tissue “i” | - | L |
| $k_{deg}$ | First-order degradation rate constant of FcRn unbound mAb within the endosomal space in moue PBPK model | 15.3 | 1/h |
| ${CL}_{up}$ | Rate of pinocytosis and exocytosis per unit endosomal space | 1.22 | L/h/L |
| $k_{on}^{FcRn}$ | Association rate constant between mAb and FcRn | 8.06E+07 | 1/M/h |
| $k_{off}^{FcRn}$ | Dissociation rate constant between mAb and FcRn | 6.55 | 1/h |
| *FcRn* | FcRn concentration in endosomal space | 4.98E-05 | M |
| FR | The fraction of FcRn-bound mAb recycles to vascular space | 0.715 | - |
| $\sigma_{i}^{V}$^a^ | Vascular reflection coefficient in tissue “i” | varied | - |
| $\sigma_{i}^{IS}$ | Lymph reflection coefficient in tissue “i’ | 0.2 | - |
| ${PS}_{i}$^b^ | MMAE permeability surface area in tissue “i” | varied | L/h |
| G | Factor multiplied by tissue plasma flow to make drug distribution instantaneous | 1000 | - |
| $R_{cap}$ | Tumor blood capillary radius | 0.008 | mm |
| $R_{krough}$ | The average distance between two capillaries | 0.075 | mm |
| $P_{ADC}$ | The permeability rate of ADC across the tumor blood vessels | 0.01 | mm/h |
| $P_{MMAE}$ | The permeability rate of MMAE across the tumor blood vessels | 0.875 | mm/h |
| $D_{ADC}$ | The diffusion rate of ADC across the tumor blood vessels | 0.00054 | mm^2^/h |
| $D_{MMAE}$ | The diffusion rate of MMAE across the tumor blood vessels | 1.04 | mm^2^/h |
| $\varepsilon_{ADC}$ | Tumor void volume for ADC | 0.24 | - |
| $\varepsilon_{MMAE}$ | Tumor void volume for MMAE | 0.44 | - |
| $k_{int}^{ADC}$ | Internalization rate of HER2-ADC complex inside the cell | 0.027 | 1/h |
| ${k_{on}}_{Antigen}^{ADC}$ | Association rate constant between ADC and HER2 | 1.25 | 1/nM/h |
| ${k_{off}}_{Antigen}^{ADC}$ | Dissociation rate constant between ADC and HER2 | 2.26 | 1/h |
| ${k_{on}}_{Tubulin}^{MMAE}$ | Secondary order association rate constant between cytoplasmic MMAE and intracellular tubulin protein | 0.00187 | 1/nM/h |
| ${k_{off}}_{Tubulin}^{MMAE}$ | First-order dissociation rate constant between MMAE-tubulin complex | 0.545 | 1/h |
| $C_{Tubulin}$ | Total tubulin concentration | 500 | nM |
| $k_{in}^{MMAE}$ | MMAE nonspecific uptake rate in cancer cell | 0.185 | 1/h |
| $k_{out}^{MMAE}$ | MMAE efflux rate from the cell | 0.046 | 1/h |

^a^$\sigma_{i}^{V}$ are 0.963 (heart), 0.875 (kidney), 0.990 (brain), 0.968 (muscle), 0.785 (skin), 0.945 (fat), 0.831 (spleen), 0.958 (pancreas), 0.957 (liver), 0.946 (bone), and 0.911 (lung).

^b^${PS}_{i}$ are 1.47 (heart), 14.2 (kidney), 0.00825 (brain), 3.16 (muscle), 0.681 (skin), 0.588 (fat), 0.457 (spleen), 0.0657 (pancreas), 49.2 (liver), 0.568 (bone), 2.47 (lung), and 0.105 (blood).

**Table S5** Partition coefficient ($K_{P})$of MMAE used in the translational PBPK model for MMAE-based ADC.

| **Tissue** | $K_{P}$ |
| --- | --- |
| Plasma | - |
| Blood | 4 (rats), 3 (monkeys), 1.34 (humans) |
| Lung | 35.3 |
| Heart | 17.9 |
| Kidney | 31.6 |
| Brain | 0.411 |
| Muscle | 1.25 |
| Bone | 1.45 |
| Skin | 1.71 |
| Fat | 2.43 |
| Spleen | 27.1 |
| Pancreas | 2.11 |
| Liver | 16.1^a^ |
| Tumor | - |

^a^The apparent Kp in the liver was adjusted for CL_int_ as liver serves as the primary eliminating organ

**Table S6** Percentage prediction error (%PE) of ADC analytes in plasma across different species

| **Analytes** | **Rats** | **Monkeys** | **Humans** |
| --- | --- | --- | --- |
| Total antibody | -16.4 | -5.91 | -0.718 |
| Conjugated MMAE | -12.4 | 2.85 | -4.45 |
| Unconjugated MMAE | -28.6 | 169 | -12.6 |

%PE calculated by $\left( 1-\frac{\mathrm{AUC}_{0-t}^{\mathrm{Observed}}}{\mathrm{AUC}_{0-t}^{\mathrm{Predicted}}} \right)\times100, when \mathrm{AUC}_{0-t}^{\mathrm{Predicted}}<\mathrm{AUC}_{0-t}^{\mathrm{Observed}}, and \left( \frac{\mathrm{AUC}_{0-t}^{\mathrm{Predicted}}}{\mathrm{AUC}_{0-t}^{\mathrm{Observed}}}-1 \right)\times100, when \mathrm{AUC}_{0-t}^{\mathrm{Predicted}}\geq\mathrm{AUC}_{0-t}^{\mathrm{Observed}}$. The two-fold overprediction and underprediction correspond to %PE of 100% and -100%, respectively.

**Table S7** Percentage prediction error (%PE) of ADC analytes in tissues in rats

| **Analytes** | **Total antibody** | **Conjugated MMAE** | **Unconjugated MMAE** |
| --- | --- | --- | --- |
| Lung | 65.7 | 77.2 | -27.0 |
| Liver | 12.8 | -18.9 | -156 |
| Heart | -68.9 | -161 | -2.71 |
| Kidney | -22.6 | 1.62 | -0.450 |
| Spleen | 160 | -4.86 | -4.29 |
| Muscle | 97.2 | 72.8 | - |
| Skin | 37.4 | 94.3 | -387 |
| Fat | 17.7 | 84.5 | -935 |
| Brain | 100 | 223 | - |
| Small intestine | 1.34 | 13.0 | -41.6 |
| Large intestine | -20.8 | -4.64 | -41.1 |
| Thymus | 95.5 | 219 | -81.2 |
| Bone marrow | -68.2 | -120 | - |

%PE calculated by $\left( 1-\frac{\mathrm{AUC}_{0-t}^{\mathrm{Observed}}}{\mathrm{AUC}_{0-t}^{\mathrm{Predicted}}} \right)\times100, when \mathrm{AUC}_{0-t}^{\mathrm{Predicted}}<\mathrm{AUC}_{0-t}^{\mathrm{Observed}}, and \left( \frac{\mathrm{AUC}_{0-t}^{\mathrm{Predicted}}}{\mathrm{AUC}_{0-t}^{\mathrm{Observed}}}-1 \right)\times100, when \mathrm{AUC}_{0-t}^{\mathrm{Predicted}}\geq\mathrm{AUC}_{0-t}^{\mathrm{Observed}}$. The two-fold overprediction and underprediction correspond to %PE of 100% and -100%, respectively; - $\mathrm{AUC}_{0-t}^{\mathrm{Observed}}$ not calculated due to limited data available.

**Table S8** Observed and model-predicted AUC_0-t_ and half-life of different ADC analytes in plasma in rats, monkeys, and humans.

| Species | Analyte | Observed AUC_0-t_ (day∙nM) | Predicted AUC_0-t_ (day∙nM) | T_last_^a^ (day) | Observed half-life (day) | Predicted half-life (day) |
| --- | --- | --- | --- | --- | --- | --- |
| Rat | Total mAb | 6474 ± 296 | 5563 | 35 | 5.64 | 5.93 |
|  | Conjugated MMAE | 8204 ± 1127 | 7301 | 14 | 2.50 | 2.40 |
|  | Unconjugated MMAE | 121 ± 40.9 | 94.2 | 14 | 1.91 | 2.39 |
| Monkey | Total mAb | 1701 ± 55.7 | 1606 | 42 | 7.47 | 5.85 |
|  | Conjugated MMAE | 2526 ± 321 | 2598 | 14 | 2.88 | 3.60 |
|  | Unconjugated MMAE | 0.795 ± 0.155 | 2.62 | 7 | 3.47 | 3.58 |
| Human | Total mAb | 1143 ± 25.6 | 1135 | 21 | 12.8 | 6.38 |
|  | Conjugated MMAE | 2951 ± 94.9 | 2825 | 21 | 5.21 | 4.07 |
|  | Unconjugated MMAE | 76.4 ± 2.63 | 67.9 | 21 | 3.94 | 4.95 |

^a^T_last_ represents the last time point of the observed PK data; therefore, model-predicted AUC_0-t_ was calculated over the same time period. Observed AUC_0-t_ values were derived by pooling all literature-reported data and applying the sparse sampling method in Phoenix WinNonlin (Version 8.4; Certara USA, Inc., 2023), reported as estimate ± SE.

**Table S9** Observed and model-predicted AUC_0-t_ of different ADC analytes in tissues in rats.

| Tissue | Analyte | Observed AUC_0-t_ (day∙nM) | Predicted AUC_0-t_ (day∙nM) | T_last_^a^  (day) |
| --- | --- | --- | --- | --- |
| Heart | Total mAb | 389 ± 34.3 | 230 | 14 |
|  | Conjugated MMAE | 952 ± 297 | 364 | 14 |
|  | Unconjugated MMAE | 272 ± 66.4 | 280 | 14 |
| Liver | Total mAb | 408 ± 37.5 | 460 | 14 |
|  | Conjugated MMAE | 865 ± 155 | 728 | 14 |
|  | Unconjugated MMAE | 626 ± 74.7 | 245 | 14 |
| Lung | Total mAb | 529 ± 81.9 | 877 | 14 |
|  | Conjugated MMAE | 785 ± 137 | 1391 | 14 |
|  | Unconjugated MMAE | 357 ± 87.6 | 454 | 14 |
| Kidney | Total mAb | 472 ± 1.77 | 385 | 14 |
|  | Conjugated MMAE | 600 ± 88.6 | 609 | 14 |
|  | Unconjugated MMAE | 492 ± 109 | 489 | 14 |
| Spleen | Total mAb | 299 ± 35.2 | 778 | 14 |
|  | Conjugated MMAE | 1287 ± 215 | 1228 | 14 |
|  | Unconjugated MMAE | 468 ± 82 | 449 | 14 |
| Skin | Total mAb | 470 ± 17.6 | 646 | 14 |
|  | Conjugated MMAE | 462 ± 76.2 | 898 | 14 |
|  | Unconjugated MMAE | 220 ± 7.76 | 45.1 | 14 |
| Fat | Total mAb | 165 ± 16.9 | 194 | 14 |
|  | Conjugated MMAE | 157 ± 16.7 | 290 | 14 |
|  | Unconjugated MMAE | 438 ± 335 | 42.4 | 14 |
| Muscle | Total mAb | 102 ± 9.35 | 200 | 14 |
|  | Conjugated MMAE | 179 | 309 | 14 |
|  | Unconjugated MMAE |  |  | 1 |
| Large intestine | Total mAb | 180 ± 5.17 | 149 | 14 |
|  | Conjugated MMAE | 245 ± 55.6 | 234 | 14 |
|  | Unconjugated MMAE | 290 ± 28.3 | 409 | 14 |
| Small intestine | Total mAb | 196 ± 10.2 | 198 | 14 |
|  | Conjugated MMAE | 276 ± 31.8 | 312 | 14 |
|  | Unconjugated MMAE | 288 ± 45.7 | 408 | 14 |
| Bone | Conjugated MMAE | 776 | 353 | 14 |
|  | Unconjugated MMAE | - | - | - |
|  | Total mAb | 410 ± 150 | 244 | 14 |
| Brain | Total mAb | 71.5 ± 2.28 | 143 | 14 |
|  | Conjugated MMAE | 59.3 | 191 | 7 |
|  | Unconjugated MMAE | - | - | - |
| Thymus | Total mAb | 192 ± 13.4 | 375 | 14 |
|  | Conjugated MMAE | 186 | 593 | 14 |
|  | Unconjugated MMAE | 768 | 424 | 14 |

^a^T_last_ represents the last time point of the observed PK data; therefore, model-predicted AUC was calculated over the same time period. -, data not available or an insufficient number of data points to calculate AUC_0-t_. Observed AUC_0-t_ values were derived by pooling all literature-reported data and applying the sparse sampling method in Phoenix WinNonlin (Version 8.4; Certara USA, Inc., 2023), reported as estimate ± SE. For data derived from a single PK profile, values are reported as estimates.

**Supplementary Appendix 1** Equations of translational PBPK model for MMAE-based ADC

***Unconjugated Drug***

**Plasma**

$$V_{plasma}\times\frac{dC_{MMAE}^{plasma}}{dt}=Q_{plasma}^{kidney}\times C_{plasma\_MMAE}^{kidney}+Q_{plasma}^{heart}\times C_{plasma\_MMAE}^{heart}+Q_{plasma}^{brain}\times C_{plasma\_MMAE}^{brain}+Q_{plasma}^{skin}\times C_{plasma\_MMAE}^{skin}+Q_{plasma}^{muscle}\times C_{plasma\_MMAE}^{muscle}+Q_{plasma}^{bone}\times C_{plasma\_MMAE}^{bone}+Q_{plasma}^{fat}\times C_{plasma\_MMAE}^{fat}+Q_{plasma}^{thymus}\times C_{plasma\_MMAE}^{thymus}+\left( Q_{plasma}^{liver}+Q_{plasma}^{spleen}+Q_{plasma}^{pancreas}+Q_{plasma}^{SI}+Q_{plasma}^{LI} \right)\times C_{plasma\_MMAE}^{liver}+Q_{plasma}^{other}\times C_{plasma\_MMAE}^{other}-Q_{plasma}^{lung}\times C_{MMAE}^{plasma}+{PS}_{BC}\times C_{MMAE}^{BC}\times\frac{f_{u,p}}{K_{P,BC}}-{PS}_{BC}\times C_{MMAE}^{plasma}\times f_{u,p}-\left( \frac{2\times P_{MMAE}\times R_{cap}}{R_{krough}^{2}} \right)\times\left( C_{MMAE}^{plasma}-\frac{C_{free_{ex_{MMAE}}}^{tumor}}{\varepsilon_{MMAE}} \right)\times V_{tumor}-\left( \frac{6\times D_{MMAE}}{R_{tumor}^{2}} \right)\times\left( C_{MMAE}^{plasma}-\frac{C_{free_{ex_{MMAE}}}^{tumor}}{\varepsilon_{MMAE}} \right)\times V_{tumor}+K_{dec}\times C_{ADC}^{plasma}\times V_{plasma}\times DAR$$

**Blood cells**

$$V_{BC}\times\frac{dC_{MMAE}^{BC}}{dt}=Q_{BC}^{kidney}\times C_{BC\_MMAE}^{kidney}+Q_{BC}^{heart}\times C_{BC\_MMAE}^{heart}+Q_{BC}^{brain}\times C_{BC\_MMAE}^{brain}+Q_{BC}^{skin}\times C_{BC\_MMAE}^{skin}+Q_{BC}^{muscle}\times C_{BC\_MMAE}^{muscle}+Q_{BC}^{bone}\times C_{BC\_MMAE}^{bone}+Q_{BC}^{fat}\times C_{BC\_MMAE}^{fat}+Q_{BC}^{thymus}\times C_{BC\_MMAE}^{thymus}+\left( Q_{BC}^{liver}+Q_{BC}^{spleen}+Q_{BC}^{pancreas}+Q_{BC}^{SI}+Q_{BC}^{LI} \right)\times C_{BC\_MMAE}^{liver}+Q_{BC}^{carcass}\times C_{BC\_MMAE}^{carcass}-Q_{BC}^{lung}\times C_{MMAE}^{BC}+{PS}_{BC}\times C_{MMAE}^{plasma}\times f_{u,p}{-PS}_{BC}\times C_{MMAE}^{BC}\times\frac{f_{u,p}}{K_{P,BC}}$$

**The Liver**

*Plasma*

$$V_{plasma}^{liver}\times\frac{dC_{plasma\_MMAE}^{liver}}{dt}=Q_{plasma}^{liver}\times C_{plasma\_MMAE}^{lung}+Q_{plasma}^{spleen}\times C_{plasma\_MMAE}^{spleen}+Q_{plasma}^{pancreas}\times C_{plasma\_MMAE}^{pancreas}+Q_{plasma}^{SI}\times C_{plasma\_MMAE}^{SI}+Q_{plasma}^{LI}\times C_{plasma\_MMAE}^{LI}-\left( Q_{plasma}^{liver}+Q_{plasma}^{spleen}+Q_{plasma}^{pancreas}+Q_{plasma}^{SI}+Q_{plasma}^{LI} \right)\times C_{plasma\_MMAE}^{liver}+{PS}_{BC}\times C_{BC\_MMAE}^{liver}\times\frac{f_{u,p}}{K_{P,BC}}-{PS}_{BC}\times C_{plasma\_MMAE}^{liver}\times f_{u,p}-Q_{plasma}^{liver}\times G\times C_{plasma\_MMAE}^{liver}\times f_{u,p}+Q_{plasma\_MMAE}^{liver}\times G\times C_{E\_MMAE}^{liver}\times f_{u,p} +K_{dec}\times C_{plasma\_ADC}^{liver}\times V_{plasma}^{liver}\times DAR$$

*Blood cells*

$$V_{BC}^{liver}\times\frac{dC_{BC\_MMAE}^{liver}}{dt}=Q_{BC}^{liver}\times C_{BC\_MMAE}^{lung}+Q_{BC}^{spleen}\times C_{BC\_MMAE}^{spleen}+Q_{BC}^{pancreas}\times C_{BC\_MMAE}^{pancreas}+Q_{BC}^{SI}\times C_{BC\_MMAE}^{SI}+Q_{BC}^{LI}\times C_{BC\_MMAE}^{LI}-\left( Q_{BC}^{liver}+Q_{BC}^{spleen}+Q_{BC}^{pancreas}+Q_{BC}^{SI}+Q_{BC}^{LI} \right)\times C_{BC\_MMAE}^{liver}+{PS}_{BC}\times C_{plasma\_MMAE}^{liver}\times f_{u,p}-{PS}_{BC}\times C_{BC\_MMAE}^{liver}\times\frac{f_{u,p}}{K_{P,BC}}$$

*Endosomal*

$$V_{E}^{liver}\times\frac{dC_{E\_MMAE}^{liver}}{dt}=Q_{plasma}^{liver}\times{G\times f_{u,p}\times C}_{plasma\_MMAE}^{liver}-2\times Q_{plasma}^{liver}\times{G\times C}_{E_{MMAE}}^{liver}+Q_{plasma}^{liver}\times{G\times C}_{IS_{MMAE}}^{liver}+K_{dec}\times{(C}_{E_{unbound_{ADC}}}^{liver}+C_{E_{bound_{ADC}}}^{liver})\times V_{E}^{liver}\times DAR+K_{deg}\times C_{E_{unbound_{ADC}}}^{liver}\times V_{E}^{liver}\times DAR$$

*Interstitial*

$$V_{IS}^{liver}\times\frac{dC_{IS\_MMAE}^{liver}}{dt}=Q_{plasma}^{liver}\times{G\times C}_{E\_MMAE}^{liver}-Q_{plasma}^{liver}\times{G\times C}_{IS\_MMAE}^{liver}-{PS}_{liver}\times C_{IS\_MMAE}^{liver}+{PS}_{liver}\times C_{cell\_MMAE}^{liver}\times\frac{f_{u,p}}{K_{P,liver}}-{CL}_{int}\times C_{IS\_MMAE}^{liver}+K_{dec}\times C_{IS\_ADC}^{liver}\times V_{IS}^{liver}\times DAR$$

*Cellular*

$$V_{cell}^{liver}\times\frac{dC_{cell\_MMAE}^{liver}}{dt}={PS}_{liver}\times C_{IS\_MMAE}^{liver}-{PS}_{liver}\times C_{cell\_MMAE}^{liver}\times\frac{f_{u,p}}{K_{P,liver}}$$

**Typical tissues**

*Plasma*

$$V_{plasma}^{i}\times\frac{dC_{plasma\_MMAE}^{i}}{dt}=Q_{plasma}^{i}\times C_{plasma\_MMAE}^{lung}-Q_{plasma}^{i}\times C_{plasma\_MMAE}^{i}+{PS}_{BC}\times C_{BC\_MMAE}^{i}\times\frac{f_{u,p}}{K_{P,BC}}-{PS}_{BC}\times C_{plasma\_MMAE}^{i}\times f_{u,p}-Q_{plasma}^{i}\times G\times C_{plasma\_MMAE}^{i}\times f_{u,p}+Q_{plasma}^{i}\times G\times C_{E\_MMAE}^{i}+K_{dec}\times C_{plasma\_ADC}^{i}\times V_{plasma}^{i}\times DAR$$

*Blood cells*

$$V_{BC}^{i}\times\frac{dC_{BC\_MMAE}^{i}}{dt}=Q_{BC}^{i}\times C_{BC\_MMAE}^{lung}-Q_{BC}^{i}\times C_{BC\_MMAE}^{i}+{PS}_{BC}\times C_{plasma\_MMAE}^{i}\times f_{u,p}-{PS}_{BC}\times C_{BC\_MMAE}^{i}\times\frac{f_{u,p}}{K_{P,BC}}$$

*Endosomal*

$$V_{E}^{i}\times\frac{dC_{E\_MMAE}^{i}}{dt}=Q_{plasma}^{i}\times{G\times f_{u,p}\times C}_{plasma\_MMAE}^{i}-2\times Q_{plasma}^{i}\times{G\times C}_{E_{MMAE}}^{i}+Q_{plasma}^{i}\times{G\times C}_{IS_{MMAE}}^{i}+K_{dec}\times(C_{E_{unbound_{ADC}}}^{i}+C_{E_{bound_{ADC}}}^{i})\times V_{E}^{i}\times DAR+K_{deg}\times C_{E_{unbound_{ADC}}}^{i}\times V_{E}^{i}\times DAR$$

*Interstitial space*

$$V_{IS}^{i}\times\frac{dC_{IS\_MMAE}^{i}}{dt}=Q_{plasma}^{i}\times{G\times C}_{endo}^{i}-Q_{plasma}^{i}\times{G\times C}_{IS\_MMAE}^{i}-{PS}_{i}\times C_{IS\_MMAE}^{i}+{PS}_{i}\times C_{cell\_MMAE}^{i}\times\frac{f_{u,p}}{K_{P,i}} +K_{dec}\times C_{IS\_ADC}^{i}\times V_{IS}^{i}\times DAR$$

*Cellular space*

$$V_{cell}^{i}\times\frac{dC_{cell\_MMAE}^{i}}{dt}={PS}_{i}\times C_{IS\_MMAE}^{i}-{PS}_{i}\times C_{cell\_MMAE}^{i}\times\frac{f_{u,p}}{K_{P,i}}$$

***Total antibody***

**Plasma**

$$V_{plasma}\times\frac{dC_{plasma}^{ADC}}{dt}=\left( Q_{plasma}^{kidney}-LF_{kidney} \right)\times C_{plasma\_ADC}^{kidney}+\left( Q_{plasma}^{heart}-LF_{heart} \right)\times C_{plasma\_ADC}^{heart}+\left( Q_{plasma}^{brain}-LF_{brain} \right)\times C_{plasma\_ADC}^{brain}+\left( Q_{plasma}^{skin}-LF_{skin} \right)\times C_{plasma\_ADC}^{skin}+\left( Q_{plasma}^{muscle}-LF_{muscle} \right)\times C_{plasma\_ADC}^{muscle}+\left( Q_{plasma}^{bone}-LF_{bone} \right)\times C_{plasma\_ADC}^{bone}+\left( Q_{plasma}^{fat}-LF_{fat} \right)\times C_{plasma\_ADC}^{fat}+\left( Q_{plasma}^{thymus}-LF_{thymus} \right)\times C_{plasma\_ADC}^{thymus}+\left( \left( Q_{plasma}^{liver}-LF_{liver} \right)+\left( Q_{plasma}^{spleen}-LF_{spleen} \right)+\left( Q_{plasma}^{pancreas}-LF_{pancreas} \right)+\left( Q_{plasma}^{SI}-LF_{SI} \right)+\left( Q_{plasma}^{LI}-LF_{LI} \right) \right)\times C_{plasma\_ADC}^{liver}+\left( Q_{plasma}^{other}-LF_{other} \right)\times C_{plasma\_ADC}^{other}-\left( Q_{plasma}^{lung}-LF_{lung} \right)\times C_{plasma}^{ADC}+LF_{lymph node}\times C_{lymph node}^{ADC}-\left( \frac{2\times P_{ADC}\times R_{cap}}{R_{krough}^{2}} \right)\times\left( C_{ADC}^{plasma}-\frac{C_{free\_ADC}^{tumor}}{\varepsilon_{ADC}} \right)\times V_{tumor}-\left( \frac{6\times D_{ADC}}{R_{tumor}^{2}} \right)\times\left( C_{ADC}^{plasma}-\frac{C_{free\_ADC}^{tumor}}{\varepsilon_{ADC}} \right)\times V_{tumor}$$

**Blood Cell**

$$V_{BC}\times\frac{dC_{BC}^{ADC}}{dt}=Q_{BC}^{kidney}\times C_{BC\_ADC}^{kidney}+Q_{BC}^{heart}\times C_{BC\_ADC}^{heart}+Q_{BC}^{brain}\times C_{BC\_ADC}^{brain}+Q_{BC}^{skin}\times C_{BC\_ADC}^{skin}+Q_{BC}^{muscle}\times C_{BC\_ADC}^{muscle}+Q_{BC}^{bone}\times C_{BC\_ADC}^{bone}+Q_{BC}^{fat}\times C_{BC\_ADC}^{fat}+Q_{BC}^{thymus}\times C_{BC\_ADC}^{thymus}+\left( Q_{BC}^{liver}+Q_{BC}^{spleen}+Q_{BC}^{pancreas}+Q_{BC}^{SI}+Q_{BC}^{LI} \right)\times C_{BC\_ADC}^{liver}+Q_{BC}^{other}\times C_{BC\_ADC}^{other}-Q_{BC}^{lung}\times C_{BC}^{ADC}$$

***Lymph Node***

$$V_{lymph node}\times\frac{dC_{lymph node}^{ADC}}{dt}=\left( 1-\sigma_{heart}^{IS} \right)\times LF_{heart}\times C_{IS\_ADC}^{heart}+\left( 1-\sigma_{kidney}^{IS} \right)\times LF_{kidney}\times C_{IS\_ADC}^{kidney}+\left( 1-\sigma_{muscle}^{IS} \right)\times LF_{muscle}\times C_{IS\_ADC}^{muscle}+\left( 1-\sigma_{skin}^{IS} \right)\times LF_{skin}\times C_{IS\_ADC}^{skin}+\left( 1-\sigma_{brain}^{IS} \right)\times LF_{brain}\times C_{IS\_ADC}^{brain}+\left( 1-\sigma_{fat}^{IS} \right)\times LF_{fat}\times C_{IS\_ADC}^{fat}+\left( 1-\sigma_{thymus}^{IS} \right)\times LF_{thymus}\times C_{IS\_ADC}^{thymus}+\left( 1-\sigma_{bone}^{IS} \right)\times LF_{bone}\times C_{IS\_ADC}^{bone}+\left( 1-\sigma_{liver}^{IS} \right)\times LF_{liver}\times C_{IS\_ADC}^{liver}+\left( 1-\sigma_{SI}^{IS} \right)\times LF_{SI}\times C_{IS\_ADC}^{SI}+\left( 1-\sigma_{LI}^{IS} \right)\times LF_{LI}\times C_{IS\_ADC}^{LI}+\left( 1-\sigma_{spleen}^{IS} \right)\times LF_{spleen}\times C_{IS\_ADC}^{spleen}+\left( 1-\sigma_{pancreas}^{IS} \right)\times LF_{pancreas}\times C_{IS\_ADC}^{pancreas}+\left( 1-\sigma_{other}^{IS} \right)\times LF_{other}\times C_{IS\_ADC}^{other}+\left( 1-\sigma_{lung}^{IS} \right)\times LF_{lung}\times C_{IS\_ADC}^{lung}-LF_{lymph node}\times C_{lymph node}^{ADC}$$

**The Liver**

*Plasma*

$$V_{plasma}^{liver}\times\frac{dC_{plasma\_ADC}^{liver}}{dt}=Q_{plasma}^{liver}\times C_{plasma\_ADC}^{lung}+\left( Q_{plasma}^{SI}-LF_{SI} \right)\times C_{plasma\_ADC}^{SI}+\left( Q_{plasma}^{LI}-LF_{LI} \right)\times C_{plasma\_ADC}^{LI}+\left( Q_{plasma}^{spleen}-LF_{spleen} \right)\times C_{plasma\_ADC}^{spleen}+\left( Q_{plasma}^{pancreas}-LF_{pancreas} \right)\times C_{plasma\_ADC}^{pancreas}-\left( \left( Q_{plasma}^{liver}-LF_{liver} \right)+\left( Q_{plasma}^{SI}-LF_{SI} \right)+\left( Q_{plasma}^{LI}-LF_{LI} \right)+\left( Q_{plasma}^{spleen}-LF_{spleen} \right)+\left( Q_{plasma}^{pancreas}-LF_{pancreas} \right) \right)\times C_{plasma_{ADC}}^{liver}-\left( 1-\sigma_{liver}^{V} \right)\times LF_{liver}\times C_{plasma_{ADC}}^{liver}-{CL}_{up}\times C_{plasma\_ADC}^{liver}+{CL}_{up}\times FR\times C_{E\_ADC}^{liver}$$

*Blood cells*

$$V_{BC}^{liver}\times\frac{dC_{BC\_ADC}^{liver}}{dt}=Q_{BC}^{liver}\times C_{BC\_ADC}^{lung}+Q_{BC}^{SI}\times C_{BC\_ADC}^{SI}+Q_{BC}^{LI}\times C_{BC\_ADC}^{LI}+Q_{plasma}^{spleen}\times C_{plasma\_ADC}^{spleen}+Q_{BC}^{pancreas}\times C_{BC\_ADC}^{pancreas}-\left( Q_{BC}^{liver}+Q_{BC}^{SI}+Q_{BC}^{LI}+Q_{BC}^{spleen}+Q_{BC}^{pancreas} \right)\times C_{BC\_ADC}^{liver}$$

*Endosomal*

$$V_{E}^{liver}\times\frac{dC_{E\_unbound\_ADC}^{liver}}{dt}={CL}_{up}\times\left( C_{plasma\_ADC}^{liver}+C_{IS\_ADC}^{liver} \right)-k_{on}^{FcRn}\times C_{E\_unbound\_ADC}^{liver}\times\left( {FcRn}_{liver}+FcRn \right)\times V_{E}^{liver}+k_{off}^{FcRn}\times C_{E\_bound\_ADC}^{liver}\times V_{E}^{liver}-K_{deg}\times C_{E\_unbound\_ADC}^{liver}\times V_{E}^{liver}$$

$$V_{E}^{liver}\times\frac{dC_{E\_bound\_ADC}^{liver}}{dt}={CL}_{up}\times C_{E\_bound\_ADC}^{liver}+k_{on}^{FcRn}\times C_{E\_unbound\_ADC}^{liver}\times\left( {FcRn}_{liver}+FcRn \right)\times V_{E}^{liver}-k_{off}^{FcRn}\times C_{E\_bound\_ADC}^{liver}\times V_{E}^{liver}$$

*Interstitial*

$$V_{IS}^{liver}\times\frac{dC_{IS\_ADC}^{liver}}{dt}=\left( 1-\sigma_{liver}^{V} \right)\times LF_{liver}\times C_{plasma\_ADC}^{liver}-\left( 1-\sigma_{liver}^{IS} \right)\times LF_{liver}\times C_{IS\_ADC}^{liver}+{CL}_{up}\times\left( 1-FR \right)\times C_{E\_bound\_ADC}^{liver}-{CL}_{up}\times C_{IS\_ADC}^{liver}$$

**Typical tissues**

*Plasma*

$$V_{plasma}^{i}\times\frac{dC_{plasma\_ADC}^{i}}{dt}=Q_{plasma}^{i}\times C_{plasma\_ADC}^{lung}+\left( Q_{plasma}^{i}-LF_{i} \right)\times C_{plasma\_ADC}^{i}-\left( 1-\sigma_{i}^{V} \right)\times LF_{i}\times C_{plasma\_AD}^{i}-{CL}_{up}\times C_{plasma\_ADC}^{i}+{CL}_{up}\times FR\times C_{E\_ADC}^{i}$$

*Blood cells*

$$V_{BC}^{i}\times\frac{dC_{BC\_ADC}^{i}}{dt}=Q_{BC}^{i}\times\left( C_{BC\_ADC}^{lung}-C_{BC\_ADC}^{i} \right)$$

*Endosomal*

$$V_{E}^{i}\times\frac{dC_{E\_unbound\_ADC}^{i}}{dt}={CL}_{up}\times\left( C_{plasma\_ADC}^{i}+C_{IS\_ADC}^{i} \right)-k_{on}^{FcRn}\times C_{E\_unbound\_ADC}^{i}\times\left( {FcRn}_{i}+FcRn \right)\times V_{E}^{i}+k_{off}^{FcRn}\times C_{E\_bound\_ADC}^{i}\times V_{E}^{i}-K_{deg}\times C_{E\_unbound\_ADC}^{i}\times V_{E}^{i}$$

$$V_{E}^{i}\times\frac{dC_{E\_bound\_ADC}^{i}}{dt}={CL}_{up}\times C_{E\_bound\_ADC}^{i}+k_{on}^{FcRn}\times C_{E\_unbound\_ADC}^{i}\times\left( {FcRn}_{i}+FcRn \right)\times V_{E}^{i}-k_{off}^{FcRn}\times C_{E\_bound\_ADC}^{i}\times V_{E}^{i}$$

*Interstitial*

$$V_{IS}^{i}\times\frac{dC_{IS\_ADC}^{i}}{dt}=\left( 1-\sigma_{i}^{V} \right)\times LF_{i}\times C_{plasma\_ADC}^{i}-\left( 1-\sigma_{i}^{IS} \right)\times LF_{i}\times C_{IS\_ADC}^{i}+{CL}_{up}\times\left( 1-FR \right)\times C_{E\_bound\_ADC}^{i}-{CL}_{up}\times C_{IS\_ADC}^{i}$$

***Tumor***

*Free total antibody in tumor*

$$\frac{dC_{free\_ADC}^{tumor}}{dt}=\left( \frac{2\times P_{ADC}\times R_{cap}}{R_{krough}^{2}} \right)\times\left( C_{ADC}^{plasma}-\frac{C_{free\_ADC}^{tumor}}{\varepsilon_{ADC}} \right)+\left( \frac{6\times D_{ADC}}{R_{tumor}^{2}} \right)\times\left( C_{ADC}^{plasma}-\frac{C_{free\_ADC}^{tumor}}{\varepsilon_{ADC}} \right)-{k_{on}}_{Antigen}^{ADC}\times\frac{C_{free\_ADC}^{tumor}}{\varepsilon_{ADC}}\times(C_{Antigen}-C_{bound\_ADC}^{tumor})+{k_{off}}_{Antigen}^{ADC}\times C_{bound\_ADC}^{tumor}$$

*Bound total antibody in tumor*

$$\frac{dC_{bound_{ADC}}^{tumor}}{dt}={k_{on}}_{Antigen}^{ADC}\times\frac{C_{free_{ADC}}^{tumor}}{\varepsilon_{ADC}}\times\left( C_{Antigen}-C_{bound_{ADC}}^{tumor} \right)-{k_{off}}_{Antigen}^{ADC}\times C_{bound_{ADC}}^{tumor}-k_{int}^{ADC}\times C_{bound_{ADC}}^{tumor}$$

*MMAE in tumor extracellular*

$$\frac{dC_{extra\_MMAE}^{tumor}}{dt}=\left( \frac{2\times P_{MMAE}\times R_{cap}}{R_{krough}^{2}} \right)\times\left( C_{MMAE}^{plasma}-\frac{C_{extra\_MMAE}^{tumor}}{\varepsilon_{MMAE}} \right)+\left( \frac{6\times D_{MMAE}}{R_{tumor}^{2}} \right)\times\left( C_{MMAE}^{plasma}-\frac{C_{extra\_MMAE}^{tumor}}{\varepsilon_{MMAE}} \right)-k_{in}^{MMAE}\times C_{extra_{MMAE}}^{tumor}+k_{out}^{MMAE}\times C_{intra_{free_{MMAE}}}^{tumor}+K_{dec}\times\left( C_{free_{ADC}}^{tumor}+C_{bound_{ADC}}^{tumor} \right)\times DAR$$

*Free MMAE in tumor intracellular*

$$\frac{dC_{intra\_free\_MMAE}^{tumor}}{dt}=k_{in}^{MMAE}\times C_{extra\_MMAE}^{tumor}-k_{out}^{MMAE}\times C_{intra\_free\_MMAE}^{tumor}-{k_{on}}_{Tubulin}^{MMAE}\times C_{intra\_free\_MMAE}^{tumor}\times(C_{tubulin}-C_{intra\_bound\_MMAE}^{tumor})+{k_{off}}_{Tubulin}^{MMAE}\times C_{intra\_bound\_MMAE}^{tumor}+k_{int}^{ADC}\times C_{bound\_ADC}^{tumor}\times DAR$$

*Bound MMAE in tumor intracellular*

$\frac{dC_{intra\_bound\_MMAE}^{tumor}}{dt}={k_{on}}_{Tubulin}^{MMAE}\times C_{intra\_free\_MMAE}^{tumor}\times\left( C_{tubulin}-C_{intra_{bound_{MMAE}}}^{tumor} \right)-{k_{off}}_{Tubulin}^{MMAE}\times C_{intra_{bound_{MMAE}}}^{tumor}$
